# Sustained STING-IRF7 signaling aggravates LPS-induced endometrial inflammation via excessive neutrophil extracellular traps generation

**DOI:** 10.3389/fimmu.2025.1671848

**Published:** 2026-01-09

**Authors:** Min Chu, Ding Ma, Zhan Song, Li Liang, Fengjuan Xing, Hongchu Bao

**Affiliations:** 1 Reproductive Medicine Center, The Affiliated Yantai Yuhuangding Hospital of Qingdao University, Yantai, China; 2 Shandong Provincial Key Medical and Health Laboratory of Reproductive Health and Genetics (Yantai Yuhuangding Hospital), Yantai, China; 3 Central Laboratory, The Affiliated Yantai Yuhuangding Hospital of Qingdao University, Yantai, China; 4 Department of Pathology, The Affiliated Yantai Yuhuangding Hospital of Qingdao University, Yantai, China

**Keywords:** CD11b, chronic endometritis, IRF7, neutrophil extracellular traps, STING

## Abstract

**Introduction:**

The stimulator of interferon genes (STING) is a central mediator of innate immune sensing and represents a critical regulator of chronic inflammation. Upon persistent infection, excessive neutrophil activation leads to the formation of neutrophil extracellular traps (NETs) that damage the tissues. However, the mechanism by which STING signaling regulates NETs formation under chronic inflammatory conditions remains poorly understood.

**Methods:**

In this study, using LPS-induced murine endometritis models in wild-type and STING-deficient mice, we demonstrated that STING deficiency significantly suppressed myeloperoxidase activity, and diminished NETs formation.

**Results:**

We identified neutrophil surface molecular CD11b as a key downstream target of STING, whose expression was transcriptionally regulated via IRF7. Furthermore, the STING-IRF7 axis was found to drive lipocalin-2 (LCN2) expression, which acted through its receptor MC4R to upregulate intracellular adhesion molecule-1 (ICAM-1), thereby facilitating neutrophil recruitment and NETosis during LPS stimulation. The role of this pathway was validated both *in vitro* using isolated neutrophils and *in vivo* using *Lcn2^-/-^* mice. Moreover, STING deficiency reprogramed the endometrial immune microenvironment by reducing inflammatory infiltration and restoring receptivity transcription factor homeobox A10 (HOXA10).

**Discussion:**

Our findings revealed a novel mechanism in which the STING-IRF7 pathway exacerbated endometrial inflammation and tissue damage by coordinately upregulating CD11b and activating the LCN2-ICAM-1 axis. Consequently, targeting the STING signaling pathway may offer a promising therapeutic strategy for chronic endometritis.

## Introduction

Chronic inflammation is a pervasive driver of pathogenesis across a spectrum of debilitating disorders, including cancer, neurodegenerative diseases, and metabolic syndromes (1). Despite its central role in human morbidity, the molecular orchestrators that perpetuate inflammatory cascades-transforming acute protective responses into chronic maladaptive states-remain poorly defined. This knowledge gap critically hampers the development of targeted therapies, leaving patients reliant on broad immunosuppressants that carry significant risks of infection and secondary comorbidities (2, 3).

Emerging evidence implicates dysregulated innate immune sensing as a linchpin of chronic inflammation (4). In particular, the cGAS-STING axis, canonically tasked with microbial DNA surveillance, has recently been implicated in sterile inflammatory pathologies through aberrant activation by self-nucleic acids and mitochondrial damage-associated molecular patterns (DAMPs) (5, 6). While STING activation exacerbates conditions like lupus and pulmonary fibrosis (7, 8), its tissue specific roles in mucosal inflammatory niches, such as the endometrium, remain enigmatic. Resolving this ambiguity is urgent: chronic endometritis (CE), characterized by persistent and subtle production of proinflammatory cytokines and leukocytes, triggered by uterine infection (9). This shifts the uterine environment from an anti-inflammatory stage to one of sustained inflammation, hampering proper embryo implantation (10), affects approximately 30% of women in unexplained infertility, particularly those with recurrent implantation failure, recurrent spontaneous abortion, and recurrent pregnancy loss (11–13), yet current empiric antibiotic regimens fail in > 40% of cases due to non-microbial drivers (14, 15). Given the high rates of persistent CE, increasing antibiotic resistance, and the possible decline in pregnancy success, there is an urgent need to explore the pathogenic mechanisms underlying chronic inflammation and to develop more targeted therapeutic strategies.

The uterine cavity is a critical mucosal interface for reproductive and immunological homeostasis, deploying neutrophils as first responders to microbial invasion (bacterial, viral or fungal) (16–18). These innate sentinels rapidly form neutrophil extracellular traps (NETs), which are chromatin scaffolds densely decorated with antimicrobial proteins, to immobilize pathogens and limit infection spread (19). However, in the context of unresolved inflammation or persistent antigen exposure, dysregulated NETosis transitions from host defense to self-injury, unleashing proteases (e.g., neutrophil elastase), reactive oxygen species (ROS), and citrullinated histones that directly compromise tissue integrity and amplify inflammatory cascades (20, 21). This duality underscores a fundamental paradox: while NETs are essential for microbial containment, their unchecked release fuels chronic tissue damage, a hallmark of conditions ranging from rheumatoid arthritis to inflammatory bowel disease. STING has been shown to enhance neutrophil migration via the NF-κB/CXCL1/2 pathway and to exacerbate neutrophil pyroptosis through the IRF3-NLRP3 axis (22, 23). Despite these findings, the specific roles of STING in neutrophil recruitment, NETs formation, and the procession of chronic endometritis remain poorly understood. Further research is needed to elucidate these mechanisms and their implications for chronic inflammatory conditions.

Here, we hypothesized that identifying keystone molecular mediators of chronic inflammation, which governed both immune cell hyperactivation and tissue dysfunction, was essential to breaking the pathogenic cycle. To investigate this, using a murine model of endometritis by inducing intrauterine infection with LPS. In this study, we discovered that NETs composed of neutrophil elastase 2 (ELA2), myeloperoxidase (MPO), and citrulline Histone H3 (citrulline R2+R8+R17; citH3), were present in the endometrium of patients with chronic endometritis. Furthermore, we found that endometrial STING exacerbated LPS-induced endometritis in mice by promoting NETs formation. Mechanistically, STING-IRF7 regulated lipocalin-2 (LCN2) facilitated neutrophil recruitment through intracellular adhesion molecule-1 (ICAM-1). Furthermore, the STING-IRF7 pathway enhanced CD11b protein expression, which promoted neutrophil activation and contributed to the formation of NETs. This cascade contributed to NETs formation during infection. Strikingly, genetic or pharmacological STING inhibition not only curtails NETs-driven damage but also restores implantation competence. By integrating transcriptomics profiling with advanced functional genomics, we dissected the STING-dependent inflammatory landscape in CE, revealing its dual role in licensing NETs formation and silencing endometrial receptivity. Our findings not only redefine STING as a tissue specific rheostat of reproductive-immune crosstalk but also nominate the IRF7-LCN2- ICAM-1 axis as an actionable target for precision anti-inflammatory therapy, offering new insights into the mechanisms underlying chronic endometritis and its treatment.

## Materials and methods

### Animals and experimental scheme

C57BL/6J *Sting*-deficient (*Tmem173^gt^*) mice were a gift from Dr. Xue-jie Yu lab and purchased from Jackson Laboratories (Ellsworth, ME, USA). C57BL/6J *Lcn2*-deficient mice were a gift from Dr. Huahua Du lab and purchased from Jackson Laboratories (Ellsworth, ME, USA). Female C57BL/6J wild-type (WT) mice were acquired from Jinan Peng Yue Experimental Animal Breeding Co., Ltd. (Jinan, China, animal batch number: 37009200017959). Mice was housed in individual ventilated cages with a 12 h light/dark cycle, 22 ± 2°C and 50-60% humidity. All experiments used age-matched (8–10 weeks old) female mice acclimated for at least 7 days prior to procedures. All animals were randomly assigned to the experimental groups using a computer-generated random number sequence to avoid selection bias. The cages for different treatment groups were intermingled and randomly repositioned within the animal facility rack on a daily basis to account for any environmental gradients. Mice from different experimental groups (e.g., LPS-treated and PBS-treated) were housed in separate cages to prevent cross-exposure and stress. The housing density was standardized at three mice per cage.

**Figure 1:**
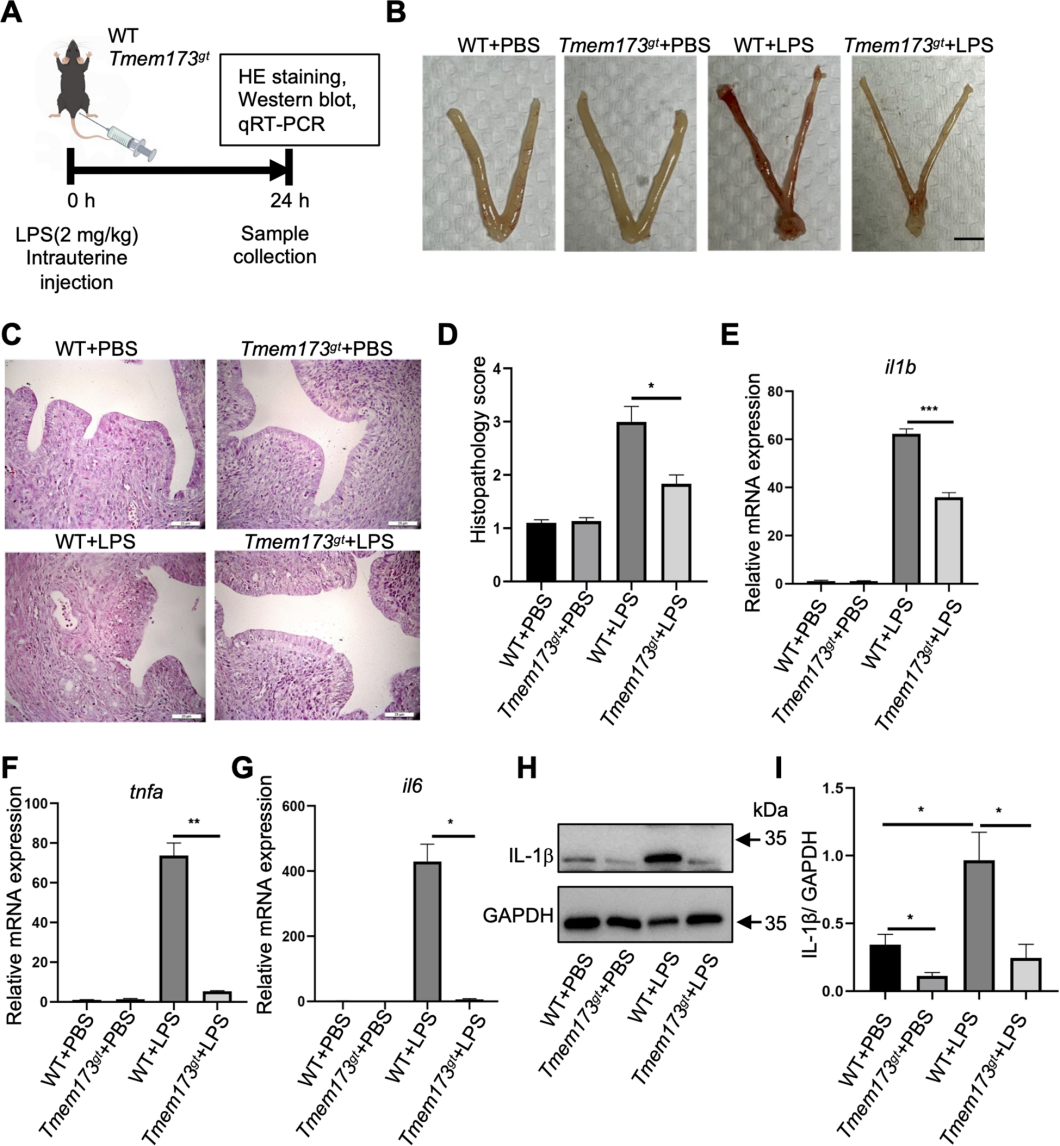
Deficiency of STING in endometrium alleviated LPS-induced endometritis. **(A)** Time axis of WT or STING-deficient mice (*Tmem173^gt^*) stimulated by LPS for 24 h to induce endometritis. **(B)** Representative macroscopic images of the uterus treatment with PBS or LPS in WT or STING-deficient mice (scale bar = 0.5 cm, n = 8). **(C)** Hematoxylin and eosin-stained of the uterine tissues in WT or STING-deficient mice in the PBS or LPS groups. Effects of STING on LPS-induced histopathological changes of uteri at 400 × magnification (scale bar = 25 μm, n=8). **(D)** Mice uteri of each group (n = 8) were processed for histological evaluation at 24 h after LPS infusion. The shedding of epithelial cells and the destroyed integrity of the endometrium in the endometritis group were observed. One-way ANOVA test was applied with *P<0.05 (WT vs. *Tmem173^gt^* in LPS group). Quantitative mRNA expression of *il1b***(E)**, *tnfa***(F)**, *il6***(G)** in endometrium of mice (n=6). One-way ANOVA test was applied with ***P<0.001 **P<0.01, *P<0.05 (WT vs. *Tmem173^gt^* in LPS group). **(H)** Representative immunoblots of the IL-1β in LPS-stimulated STING-deficient mice (*Tmem173^gt^*) endometrium tissues, compared with WT-LPS stimulated mice. The GAPDH was used as a loading control. **(I)** Quantification of the amount of IL-1β in the four groups (n=5). One-way ANOVA test was applied with *P<0.05 (WT vs. *Tmem173^gt^* in PBS group), *P<0.05 (WT vs. *Tmem173^gt^* in LPS group).

**Figure 2:**
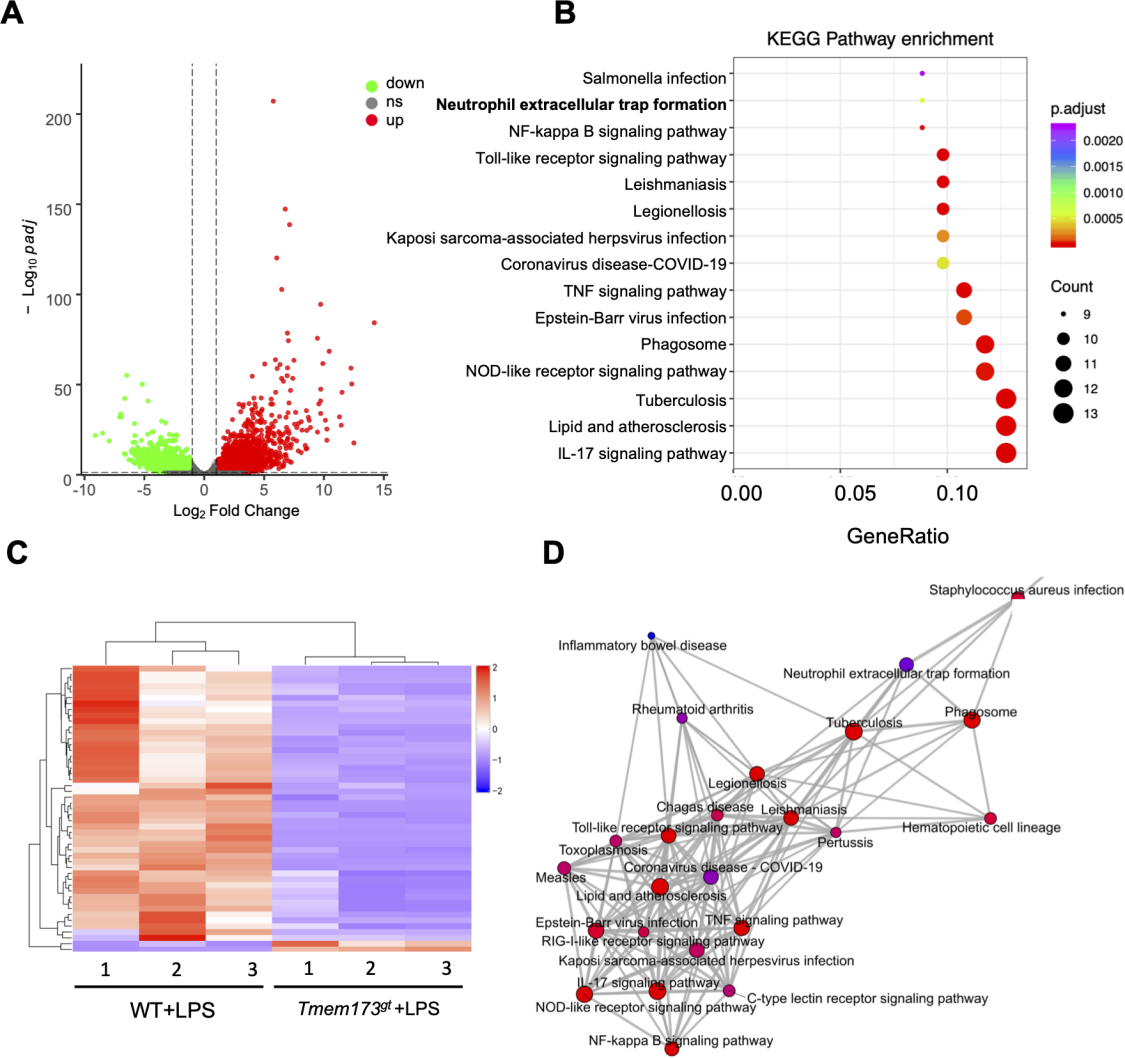
KEGG pathway enrichment analysis of DEGs regulated by STING in endometrium. **(A)** Volcano plot of up- or down-regulated differential expression genes among WT and STING-deficient mice (*Tmem173^gt^*) stimulated with LPS performed with DESeq2 (n=3). **(B)** KEGG pathway analysis of differential expression genes between WT and STING-deficient mice (*Tmem173^gt^*) stimulated with LPS groups. **(C)** Heatmap depicting a z-score hierarchical clustering based on Euclidean distance measure method for top pathway genes expressed across the WT and STING-deficient mice (*Tmem173^gt^*) infected by LPS for 24 h (n=3). **(D)** Pathway-act network showing the interaction of differential KEGG pathways.

For LPS-induced endometritis, mice were anesthetized with isoflurane (2-3%), and 50 μL of LPS (2 mg/mL, HY-D1056, MedChemExpress, USA) or PBS (vehicle) was intrauterinely injected bilaterally using a 30-gauge microsyringe (24). At 24 h post-injection, mice were euthanized via intraperitoneal pentobarbital sodium (5%, 150 mg/kg) and uterine tissues were harvested for analysis (25, 26) (**Figure 1A**). To detect the effect of STING on endometrial receptivity, mice received bilateral intrauterine LPS (2 mg/mL) or PBS injections once daily for seven consecutive days (27, 28). Uterine tissues were collected on 8 days. For fertility studies, LPS- or PBS-treated female mice were cohoused with WT males (1:1 ratio) at day 5 post-coitum (dpc). Successful mating was confirmed by vaginal plug detection. Implantation sites were quantified at day 5 dpc via uterine dissection (**Figure 2A**). The animal experiments in the study were conducted in accordance with institutional guidelines for the care and use of laboratory animals and protocols by the Medicine Ethics Committee of the Yantai Yuhuangding Hospital. The Medicine Ethics Committee of the Yantai Yuhuangding Hospital (Yantai, China) has approved the present study (No. 2021-434).

### Drug administration

STING agonist DMXAA (HY-10964, MedChemExpress, USA) were diluted in 5% DMSO and 95% saline. The WT mice and *Lcn2^-/-^* mice were injected intraperitoneally with 25 mg/kg DMXAA or saline for 3 h (29, 30). Then, the mice were administered an intrauterine injection of 50 μL LPS (2 mg/mL in sterile PBS). Mice from each group were sacrificed 24 h after LPS injection.

### Patients and sample collection

The cohort included woman (age 21-40, BMI 18.5–30 kg/m^2^) planned for IVF/ICSI treatment who undergo a diagnostic hysteroscopy in preparation of their treatment; The exclusion criteria in this study were the presence of a hydrosalpinx, uterine malformation, intrauterine adhesions, submucosal fibroids or acute and chronic infectious diseases, autoimmune diseases, diabetes, cardiovascular disease, thyroid disease and mental illness. Endometrial tissues (including negative controls, and CD138+ ≥5 chronic endometritis samples) were collected during the luteal phase using a Pipelle sampler. The tissue collected was rinsed thoroughly for 5 minutes in DNase/RNase-free water. The collected tissue was immersed into 10% neutral buffered formalin for overnight fixation at room temperature and subsequently embedded into paraffin wax for histopathologic assessment. This work was approved by the Medicine Ethics Committee of the Yantai Yuhuangding Hospital (Research Ethics Committee reference: 2021-434), and written informed consent was obtained from all participants. This study adheres to the tenets of the World Medical Association Declaration of Helsinki.

### Hematoxylin and eosin staining

Uterine tissues were fixed in 4% paraformaldehyde at room temperature for 72 h, followed by dehydration through a graded ethanol series (70%, 80%, 90% and 100%). Dehydrated tissues were embedded in paraffin and serial 4 μm-thick sections were cut using a rotary microtome. Sections were stained with hematoxylin and eosin. All images were obtained using an epifluorescence microscope (LEICA DMLB2). Endometritis severity was scored blindly by two independent pathologists using a validated 4-point scale following criteria: 0 (no histopathological changes), 1 (minimal, a subtle histopathological change that barely exceeded the normal range), 2 (mild, the histopathological change was present), 3 (moderate, the histopathological change was pronounced), and 4 (severe, the histopathological change was pronounced and significant) (31, 32).

### Immunohistochemistry staining

Paraffin-embedded uterine sections (4 μm) were deparaffinized in xylene and rehydrated through graded ethanol (100%, 95%,80%,70%) to distilled water. Endogenous peroxidase activity was quenched with 3% H_2_O_2_ in methanol for 15 min at room temperature (RT). Heat-induced epitope retrieval was performed in 10 mM sodium citrate buffer using a microwave (700 W, 15min). Sections were cooled to RT and rinsed in PBS. Non-specific binding was blocked with 3% bovine serum albumin in PBS for 1h at RT. Sections were incubated overnight at 4°C with the following primary antibodies diluted in blocking buffer: MPO (1:200, 22225-1-AP, Proteintech, China), CD11b (MAC-1) (1:100, AB133357, Abcam, USA), ELA2 (1:1000, 27642-1-AP, Proteintech, China), Ly-6G (1:100, 87048T; Cell Signaling Technology, USA). The secondary antigens used were biotinylated goat anti-rabbit (1:200, SA00004-2, Proteintech, China) and incubated for 1 h at room temperature. Signal amplification was achieved using DAB kit. Reactions were terminated by rinsing in distilled water. Sections were counterstained with Mayer’s hematoxylin for 1 min, dehydrated through ethanol-xylene, and mounted with Permount. Images were acquired using a Leica DMLB2 epifluorescence microscope and a DFC450 CCD camera.

### Immunofluorescence staining

Immunofluorescence staining to observe the expression of NETosis markers was performed on formalin-fixed paraffin-embedded tissues utilizing the following primary antibodies: rabbit polyclonal anti-MPO (1:100, 22225-1-AP, Proteintech, China), and rabbit polyclonal anti-histone H3 (citrulline R2+R8+R17) (1:100, AB281584, Abcam, USA). The expression of IL-17C was detected using rabbit polyclonal anti-IL17C (1:50, A10587, ABclonal, China). The following secondary antibodies were used: CoraLite594-conjugated Goat anti-rabbit IgG (H+L) (1:400; SA00013-4; Proteintech, China), Alexa Fluor 488-conjugated goat anti-rabbit IgG (1:200, D110061, BBI, China). The color was read and interpreted using a fluorescence confocal microscope (Axio Observer7, Carl Zeiss Microscopy, GER). A DAPI staining solution (C1005, Beyotime, China) was used for nuclear staining. The Medicine Ethics Committee of the Yantai Yuhuangding Hospital (Yantai, China) has approved the present study (No. 2021-434). Clinical tissue samples were conducted in accordance with the guidelines outlined in the Declaration of Helsinki. Written informed consent was obtained from all participants at the time of presentation for hysteroscopy treatment.

### Transcriptomics sequencing and data analysis

For LPS-induced endometritis, uterine tissues were collected from two groups: LPS-treated STING-deficient mice and LPS-treated WT mice. Each group included three biological replicates to ensure statistical robustness. Transcriptomic profiling was performed by Wekemo Tech Group Co. Ltd. (Shenzhen, China) on an Illumina HiSeq v2 platform. After filtering the raw data, the sequences were aligned to the mouse genome “Mus musculus (house mouse) (Ensembl) (accessed on May 9, 2022)” using the HISAT2 software (2.2.1). Genes with |log2 (fold change, Fc)| > 1 and a significant *p*-value less than 0.05 as evaluated by DESeq2 (1.26.0) were assigned as DEGs between the two groups. Gene Ontology (GO) and Kyoto Encyclopedia of Genes and Genomes (KEGG) pathway analysis were performed using the Functional Annotation Bioinformatics Microarray Analysis (DAVID) database (https://davidbioinformatics.nih.gov/). All raw and processed transcriptomic datasets, including differentially expressed genes, pathway enrichment analyses, and visualization outputs, have been archived in **Supplementary Data 1** through 5 (**Supplementary Data 1–5**).

### Neutrophil isolation and stimulation

Neutrophils were isolated from the bone marrow using the mouse neutrophil isolation kit (P8550, Solarbio, China). Freshly isolated neutrophils were suspended in RPMI-1640 (Gibco, MA) and seeded in 12-well glass-bottomed plates in a 5% CO_2_ atmosphere at 37°C for 30 min before stimulation. Following incubation with the STING agonist cGAMP (20 mM, T10065, targetMol, China) and STING inhibitor C-176 (1 μM, T5154, targetMol, China) or H-151 (1 μM, HY-112693, MedChem Express, USA) or vehicle (saline containing 0.5% DMSO) for 30 min after incubation with 1μg/mL LPS at 37°C for 2.5h.

### Flow cytometry

After stimulation with LPS, the neutrophils were then fixed in 2% paraformaldehyde, blocked for 30 min with 2% bovine serum albumin (BSA, Serviers) at 37°C and, without a permeabilization step, incubated sequentially with the antibodies, including APC CD11b (1:200, 101212, Biolegend, USA), FITC CD45 (1:200, 103108, Biolegend, USA), and PE Ly6G (1:200, 127608, Biolegend, USA) antibodies (33). The primary anti-histone H3 antibody (citrulline 2,8,17, ab5103, Abcam, USA) was stained at 1:200 dilution, CL594-conjugated secondary antibody (SA00013-4, Proteintech, China) was stained at 1:300 dilution. Each incubation was followed by a wash with 2% BSA and centrifugation at 12,000 rpm at 4°C for 30 min (34). A minimum of 10,000 events per condition were acquired in duplicate on a Thermo Fisher Attune NxT flow cytometer. Neutrophils were identified as CD45^+^CD11b^+^Ly6G^+^ cells. The mean fluorescence intensity (MFI) of CD11b and citH3 on neutrophils was measured (35).

### Cell culture and transfection

Mouse endometrial epithelial cells (EECs)were enzymatically isolated from the uterine tissues of the mice as previously described (36). Mouse EECs were seeded at a density of 5× 10^5^ per well in 12-well plates pre-coated with collagen I peptide. Cells were maintained in complete DMEM/F12 medium supplemented with charcoal-stripped 10% FBS in a 5% CO_2_ incubator at 37°C expansion culture for subsequent experiments.

The human endometrial epithelial cell line Ishikawa was obtained from the American Type Culture Collection (ATCC, Manassas, VA, USA). Ishikawa cells at a density of 5× 10^5^ per well in 12-well plates were maintained in DMEM. All cell-conditioned media were supplemented with 10% (v/v) fetal bovine serum (FBS), 100 IU/mL penicillin, and 100 mg/mL streptomycin. The cells were cultured at 37°C under 5% CO_2_ in humidified air, according to standard procedures. The medium was renewed every 2–3 days.

For co-culture experiment, neutrophils (1 × 10^5^ cells) were seeded in the upper chamber of a 12-well Tran-swell plate, and mouse endometrial epithelial cells (EECs) (1 × 10^5^ cells) were cultured in the lower chamber (19). Following incubation with the ZINC00640089 (10 mM, T72968, TargetMol, China) or vehicle (saline containing 0.5% DMSO) in the lower chamber for 30 min after incubation with 10 mg/mL LPS at 37°C for 2.5h.

For cellular depletion assays, siRNA specific for IRF7 (si-IRF7) and a nontarget siRNA control (si-NC) were obtained from Ribobio (Guangzhou, China). IRF7 overexpression was achieved using pCMV-IRF7 plasmid (Miaoling Biology, Wuhan, Chian), with pCMV-vector serving as the control. At 48 h post-transfection, the EECs were subjected to LPS (1μg/mL) stimulation for 24 h prior to lysed for protein extraction.

### Quantitative real-time PCR assay

Total RNA was extracted from uterine tissues and primary cells using SparkZol Reagent (AC0101, SparkJade, China) according to the manufacturer’s protocol. The concentration and purity of the extracted RNA were measured, with typical yields yielding concentrations in the range of 200–500 ng/μL. Relative mRNA expression levels were determined using the HiScript IV RT SuperMix for qRT-PCR (+gDNA wiper) (R423-01, Vazyme, China), and cDNAs was run on an FTC-3000P (Funglyn Biotech, Canada) using ChamQ SYBR qRT-PCR Master Mix (Q311-02, Vazyme, China). The primers used were listed in **Table 1**. The relative expression levels were calculated using the 2^-ΔΔCT^ method and GAPDH was used as an endogenous control to normalize the data.

**Table 1:** Primer sequences for the targeted genes in qRT-PCR.

Gene	Accession number	Sequence (5’→3’)
*il1b* (mouse)	NM_008361.1	F: GGACCTTCCAGGATGAGGACA R: GTTCATCTCGGAGCCTGTAGTG
*tnfa* (mouse)	NM_013693.1	F: GGTGCCTATGTCTCAGCCTCTT R: GCCATAGAACTGATGAGAGGGAG
*il6* (mouse)	NM_031168.1	F: TACCACTTCACAAGTCGGAGGC R: CTGCAAGTGCATCATCGTTGTTC
*irf7* (mouse)	NM_016850.1	F: CCTCTGCTTTCTAGTGATGCCG R: CGTAAACACGGTCTTGCTCCTG
*vdr* (mouse)	NM_009504.2	F: GCTCAAACGCTGCGTGGACATT R: GGATGGCGATAATGTGCTGTTGC
*cebpb* (mouse)	NM_009883.1	F: CAACCTGGAGACGCAGCACAAG R: GCTTGAACAAGTTCCGCAGGGT
*24p3r* (mouse)	NM_021551.1	F:CCTTGTCTCTAAGGACTGGCGA R: ATCTGCCGCTTCACTATCAGCC
*megalin* (mouse)	NM_001081088	F: CCAATGGACTCACTCTGGACCT R: GAATGGAAGGCAGTGCTGATGAC
*mc4r* (mouse)	NM_016977.1	F: CGAGGTGTTTGTGACTCTGGGT R: AACGCTCACCAGCATATCTGCC
*lcn2* (mouse)	NM_008491.1	F: ATGTCACCTCCATCCTGGTCAG R: GCCACTTGCACATTGTAGCTCTG
*hoxa10* (mouse)	NM_008263.1	F: CTGTCTCCAGCCCCTTCAGAAA R: TCTGGTGCTTCGTGTAAGGGCA
*Itgb3* (mouse)	NM_016780.1	F: GTGAGTGCGATGACTTCTCCTG R: CAGGTGTCAGTGCGTGTAGTAC
*il17c* (mouse)	NM_145834.1	F: AGGTGCTGGAAGCTGACACTCA R: TCCACGACACAAGCATTCTGCC
*cxcl2* (mouse)	NM_009140.1	F: CATCCAGAGCTTGAGTGTGACG R: GGCTTCAGGGTCAAGGCAAACT
*cxcl5* (mouse)	NM_009141.1	F: CCGCTGGCATTTCTGTTGCTGT R: CAGGGATCACCTCCAAATTAGCG
*gapdh* (mouse)	BC096042.1	F: CATCACTGCCACCCAGAAGACTG R: ATGCCAGTGAGCTTCCCGTTCAG
*itgam* (human)	NM_001082960.1	F: TACTTCGGGCAGTCTCTGAGTG R: ATGGTTGCCTCCAGTCTCAGCA
*gapdh* (human)	NM_014364.1	F: GCCATCAAGGAGGCTGTAAAAGC R: GGTATCACCGAGGAAGTCCGTA

### Protein extraction and Western blotting analysis

Mouse tissues were lysed with RIPA lysis solution (EA0002, SparkJade, China) supplemented with 1% PMSF and centrifuged at 4°C and 12,000 rpm for 10 min to harvest total protein, as described by the manufacturer. Protein concentrations of the lysates were determined using a BCA Protein Quantification kit (E112-01, Vazyme, China) according to the manufacturer’s instructions. After extracting uterine tissue proteins, NETs-related proteins were separated using an 8% One-Step PAGE Gel Fast Preparation Kit (E302-C1, Vazyme, China) and 12% One-Step PAGE Gel Fast Preparation Kit (E304-C1, Vazyme, China) and transferred to nitrocellulose membranes. After blocking with 5% non-fat milk for 1 h, cells were incubated with the following antibodies: IL-1β (1:1000, 26048-1-AP, Proteintech, China), citH3 (1:1000, AB281584, Abcam, USA), ELA2 (1:1000, 27642-1-AP, Proteintech, China) and MPO (1:1000, 22225-1-AP, Proteintech, China), CD11b (MAC-1) (1:1000, AB133357, Abcam, MA, USA), IRF7(1:1000, Cat. no. 22392-1-AP, Proteintech, Wuhan, China), CEBPB (1:1000, D155298; BBI, China), and VDR (1:1000, D151709, BBI, China), and GAPDH (1:1000, 60004-1-AP; Proteintech, China) overnight at 4°C and washed in Tris-buffered saline (TBST) in triplicate for 5 min. The nitrocellulose membranes were then incubated with secondary antibody (1:1000, SA00001-2; Proteintech, China) for 60 min at room temperature. Finally, the mixture was exposed and analyzed using a chemiluminescence imaging system (ChemiScope6200 Touch; Clinx Science Instruments Co., Ltd.). The grayscale value of each band was measured using the ImageJ software (version 1.8.0; National Institutes of Health).

### Myeloperoxidase activity assay

Uterine tissues were homogenized and were used to test MPO activity was measured using an MPO Activity Assay Kit (BC5710, Solarbio, China). The assay was performed according to the manufacturer’s instructions.

### Statistics

All data were expressed as mean ± SEM and were analyzed using SPSS (version 22.0, IBM, Chicago, IL, USA). Significance was analyzed using one-way ANOVA analysis of variance, following by Tukey’s *post hoc* test and Student’s unpaired *t* test using GraphPad Prism software (version 8.0). *p*<0.05 indicated a significant difference.

## Results

### STING deficiency mitigated LPS-induced endometrial inflammation and tissue damage in mice

To investigate the role of STING in chronic endometritis, a murine endometritis model induced with LPS was established (**Figure 1A**). Wide-type (WT) mice challenged with LPS exhibited severe macroscopic pathological features, including pronounced edema and vascular hyperemia in the uterine tissue. In contrast, STING-deficient mice displayed markedly attenuated pathological symptoms (**Figure 1B**), suggesting a protective effect of STING deficiency against LPS-induced tissue damage. Histopathological analysis via H&E staining further revealed prominent neutrophils infiltration, epithelial cell apoptosis, and luminal epithelial shedding in LPS-treated WT uteri, all of which were significantly alleviated in STING-deficient mice (**Figures 1C, D**), indicating that STING signaling plays a critical role in driving endometrial inflammation and tissue injury. To further characterize the inflammatory response, we measured the expression levels of key pro-inflammatory cytokines in uterine tissues. LPS challenge triggered a robust upregulation of *il1b* (**Figure 1E**)*, tnfa* (**Figure 1F**), and *il6* (**Figure 1G**) in WT mice, confirming the successful induction of endometritis. Strikingly, STING deficiency suppressed the expression of these cytokines, highlighting the central role of STING in amplifying the inflammatory cascade during endometritis. To validate these findings at the protein level, IL-1β protein levels in uterine tissues were quantified. Consistent with the mRNA data, IL-1β protein levels were significantly reduced in STING-deficient uteri compared to WT controls post-LPS challenge (**Figures 1H, I**). These further underscores the critical involvement of STING in amplifying endometrial inflammation and suggests that STING deficiency mitigates the inflammatory response by downregulating key pro-inflammatory mediators.

### STING deficiency suppressed NETs formation in endometritis

To explore the specific regulation genes by STING in endometrium, endometrial tissues from LPS-treated WT and STING-deficient mice were collected and subjected to RNA sequencing (**Supplementary Figure 1A**). Comparative analysis revealed significant differences in gene expression profiles between the STING-deficient endometritis group and the WT control group (**Supplementary Figure 1B**). Using stringent criteria absolute value log2 (Fold Change) > 1 and Padj (adjusted *p*)<0.05, we identified 1449 upregulated and 1677 downregulated genes in STING-deficient mice compared to WT controls (**Figure 2A**). To elucidate the biological pathways influenced by STING deficiency, we conducted KEGG pathway enrichment analysis on the differentially expressed genes. The results demonstrated that STING depletion predominantly affected several keys signaling pathways, including the NF-κB signaling pathway, Nod-like receptor signaling pathway, neutrophil extracellular trap formation, and cytokine activity pathways (**Figure 2B**). Among the top 50 detected DEGs, majority of the genes (96%, 48 out of 50) were found to be downregulated in LPS-stimulated STING-deficient group (**Figure 2C**). Notably, Pathway-act network revealed that neutrophil extracellular trap formation was significantly decreased in the STING-deficient group (**Figure 2D**), suggesting a direct role for STING in regulating NETs during endometritis.

**Figure 3:**
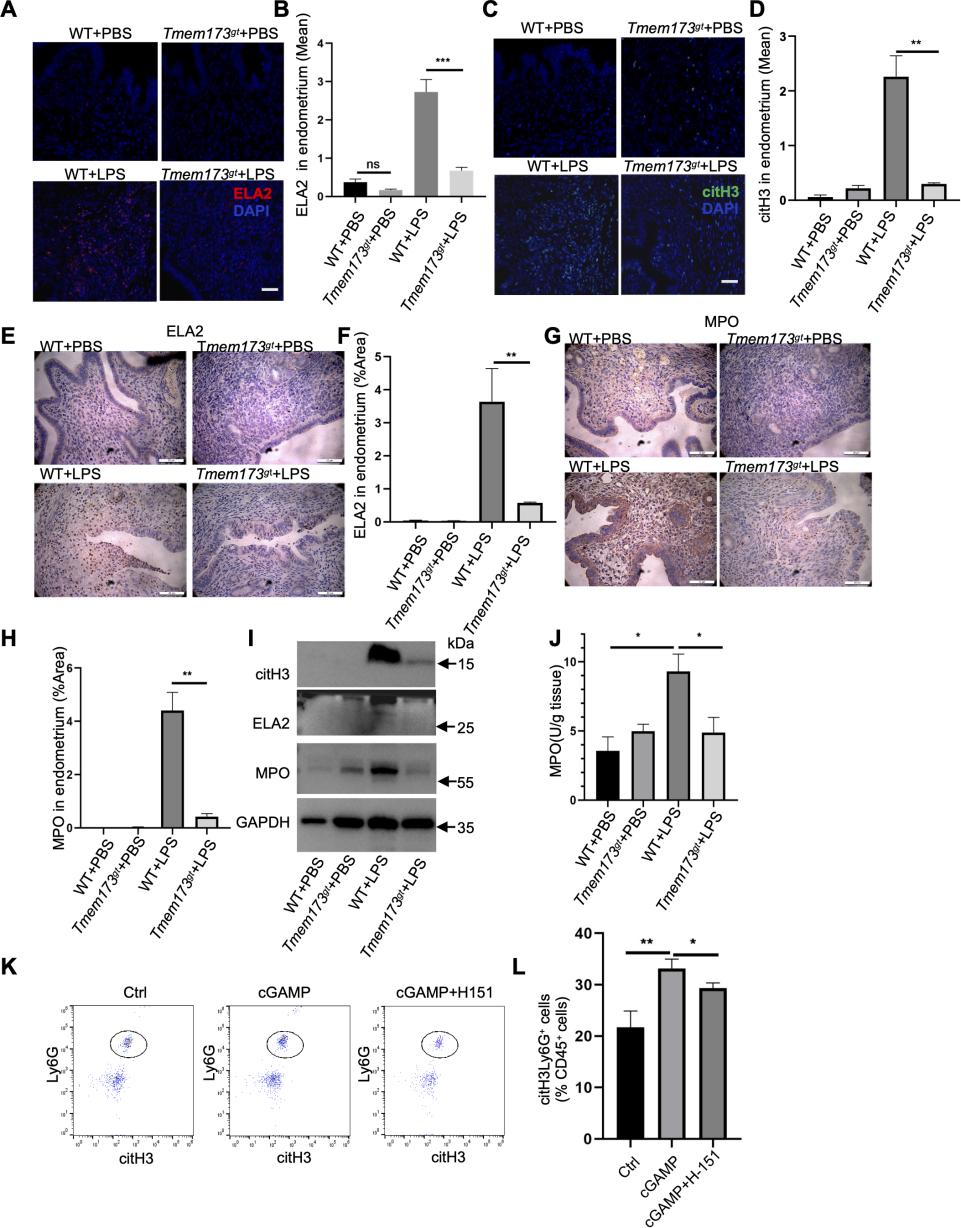
STING deficiency suppressed NETs formation in endometritis. **(A)** Representative images of ELA2 (red) in LPS-treatment *Tmem173^gt^* mice endometrial tissues or control for 24 h at 400 × magnification (scale bar = 25 μm). The DNA was staining with DAPI. **(B)** Quantification of ELA2-positive cells in the endometrial tissues (n=10). One-way ANOVA test was applied with ***P<0.001 (WT vs. *Tmem173^gt^* in LPS group). **(C)** Representative images of citH3 (green) in LPS-treatment Tmem173^gt^ mice endometrial tissues or control for 24 h at 400 × magnification (scale bar = 25 μm). The DNA was staining with DAPI. **(D)** Quantification of citH3 positive cells in the endometrial tissues (n=10). One-way ANOVA test was applied with **P<0.01 (WT vs. *Tmem173^gt^* in LPS group). **(E)** Representative immunohistochemical staining of ELA2 in endometrial tissues of chronic endometritis or control at 400 × magnification (scale bar = 25 μm). **(F)** The area of ELA2 were quantified (n=8). One-way ANOVA test was applied with **P<0.01 (WT vs. *Tmem173^gt^* in LPS group). **(G)** Representative immunohistochemical staining of MPO in endometrial tissues of chronic endometritis or control at 400 × magnification (scale bar = 25 μm). **(H)** The area of MPO were quantified (n=8). One-way ANOVA test was applied with **P<0.01 (WT vs. *Tmem173^gt^* in LPS group). **(I)** Representative immunoblots of citH3, ELA2 and MPO in LPS-treatment *Tmem173^gt^* mice endometrial tissues or control endometrium tissues. **(J)** MPO concentration in endometrial tissues of LPS-treatment *Tmem173^gt^* mice endometrial tissues or control (n=6). One-way ANOVA test was applied with *P<0.05 (WT vs. *Tmem173^gt^* in PBS group), *P<0.05 (WT vs. *Tmem173^gt^* in LPS group). **(K)** Detection of NETs formed by flow cytometry in mice bone marrow neutrophils stimulated with STING agonis cGAMP and or STING inhibitor H-151. **(L)** Quantification of citH3^+^cells on neutrophils stimulated with STING agonis cGAMP and or STING inhibitor H-151 by flow cytometry. Representative flow data showing citH3^+^ cells defined as NETs. One-way ANOVA test was applied with **P<0.001, * *P*<0.05.

To validate the role of STING in the pathogenesis of endometritis and the formation of NETs, the levels of key NETs markers, ELA2 (**Figures 3A, B**) and citH3 (**Figures 3C, D**) were assessed in both WT and STING-deficient intrauterine mice following LPS injection using immunofluorescence staining. Strikingly, STING-deficient mice exhibited a significant reduction in NETs production compared to WT mice, suggesting that STING signaling is essential for the induction of NETs in the context of endometritis. To further investigate the underlying mechanisms, we preformed immunohistochemical analysis on endometrial tissues. The results demonstrated that the expression levels of ELA2 (**Figures 3E, F**) and MPO (**Figures 3G, H**), two critical markers associated with neutrophil activation and NETs formation, were markedly elevated in the WT endometritis group compared to STING-deficient mice. This finding indicates that STING deficiency attenuates neutrophil-driven inflammatory responses in endometrial tissues. To validate these observations at the molecular level, we conducted Western blotting to quantify the expression of NETs-associated proteins, including citH3, ELA2, and MPO. Consistent with our earlier findings, the expression of these proteins was significantly elevated in LPS-stimulated WT uterine tissues but reduced in STING-deficient mice (**Figure 3I**), further supporting the critical role of STING in promoting NETs formation and neutrophil activation during endometritis. Given that MPO is predominantly expressed in neutrophils and serves as a marker for neutrophil infiltration, MPO activity was measured using MPO assay kits. The findings revealed a substantial increase in MPO activity in the LPS-treated uterine tissue of WT mice, indicative of enhanced neutrophil infiltration and activation. In contrast, STING-deficient mice exhibited diminished MPO levels in response to LPS stimulation (**Figure 3J**), suggesting that STING deficiency impairs neutrophil recruitment and activation in the context of endometritis. To investigate whether STING directly contributed to NETs formation, we treated isolated bone marrow neutrophils with STING agonist cGAMP and or H-151, and measured NETs marker citH3 expression using multicolor flow cytometry without a permeabilization step to exclude the citH3 expressed in neutrophils cytoplasm. The result showed that cGAMP had positive effect on citH3 expression; in contrast, H-151 significantly downregulated citH3 expression (**Figures 3K, L**), which demonstrated that STING had positive effect on NETs formation.

### CD11b was regulated by STING-IRF7 pathway in endometrium

**Figure 4:**
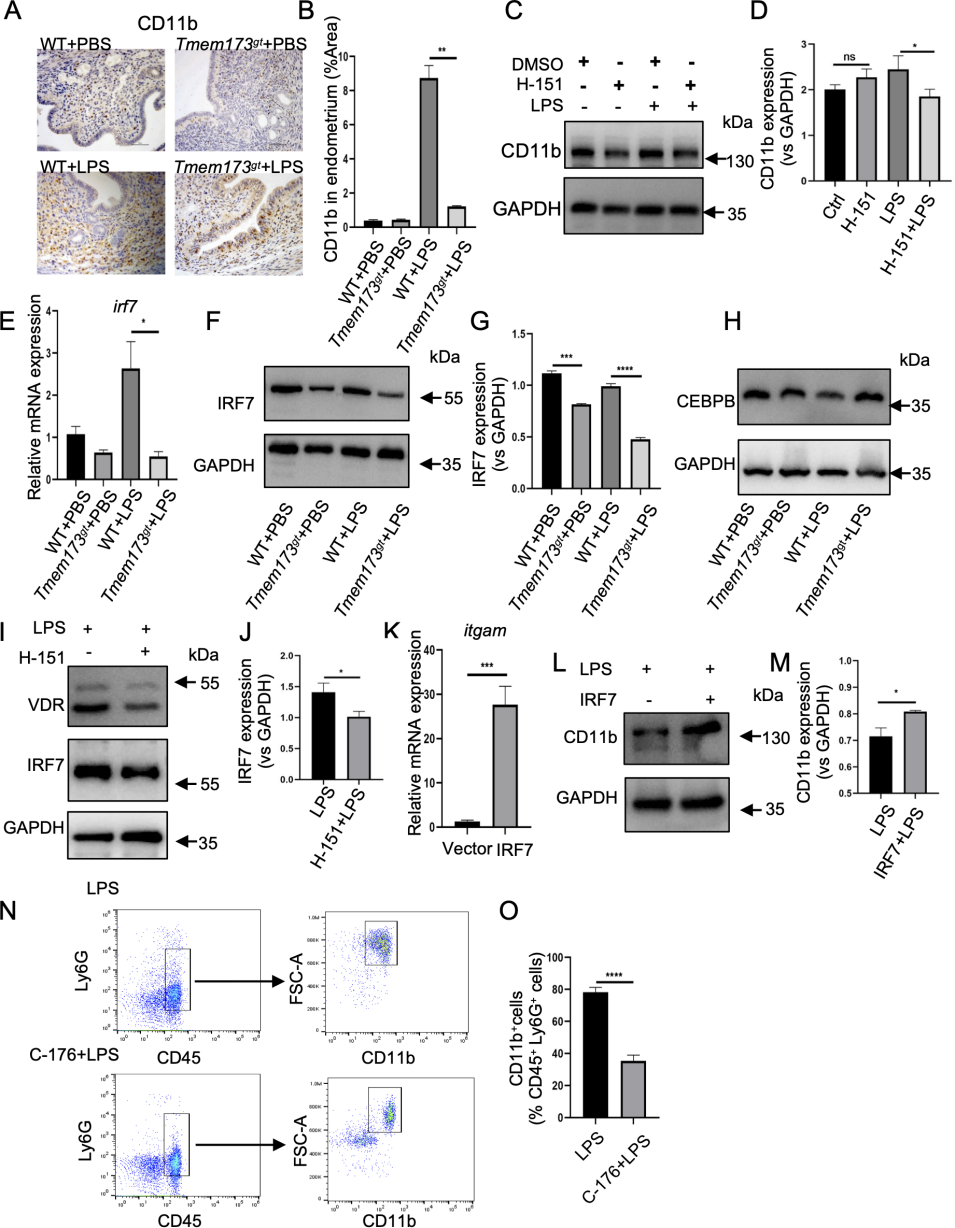
CD11b was regulated by STING-IRF7 pathway in endometrium. **(A)** Representative immunohistochemical staining of CD11b in endometrial tissues of WT or STING-deficient mice (*Tmem173^gt^*) infected by LPS for 24 h at 400 × magnification (scale bar = 25 μm). **(B)** The area of CD11b were quantified (n=6). One-way ANOVA test was applied with **P<0.01 (WT vs. *Tmem173^gt^* in LPS group). **(C)** STING inhibitor H-151was used to pretreat Ishikawa cell with or without LPS infected for 24 h, then the protein expression of CD11b was detected by Western blotting. **(D)** Quantification of the amount of CD11b in the four groups (n=5). One-way ANOVA test was applied with *P<0.05 (LPS vs. H-151+LPS group). **(E)** Quantitative mRNA expression of *irf7* in endometrium of mice (n=6). One-way ANOVA test was applied with *P<0.05 (WT vs. *Tmem173^gt^* in LPS group) **(F)** Representative immunoblots of IRF7 in LPS-stimulated STING-deficient mice (*Tmem173^gt^*) endometrium tissues, compared with WT-LPS stimulated mice. **(G)** Quantification of the amount of IRF7 in the four groups (n=4). One-way ANOVA test was applied with ****P<0.0001(WT vs. *Tmem173^gt^* in LPS group). **(H)** Representative immunoblots of CEBPB in LPS-stimulated STING-deficient mice (*Tmem173^gt^*) endometrium tissues, compared with WT-LPS stimulated mice. **(I)** Representative immunoblots of IRF7 in STING inhibitor H-151 pretreat Ishikawa cell. **(J)** Quantification of the amount of IRF7 in the two groups, and *t* test was applied with *P<0.05 (LPS vs. H-151+LPS group). **(K)** Quantitative mRNA expression of *itgam* with IRF7 overexpression in Ishikawa cells, and *t* test was applied with ***P<0.001 (LPS vs. H-151+LPS group). **(L)** Representative immunoblots of CD11b with the transfection of IRF7 in Ishikawa cells. **(M)** Quantification of the amount of CD11b in the two groups, and *t* test was applied with *P<0.05 (LPS vs. IRF7+LPS group). **(N)** Detection of CD11b expression by flow cytometry in mice bone marrow neutrophils stimulated with STING inhibitor C-176. **(O)** Quantification of CD11b expression on Ly6G^+^ CD45^+^ cells in mice bone marrow neutrophils stimulated with STING inhibitor C-176., and *t* test was applied with ****P<0.0001. Independent experiments are repeated at least three times.

To further elucidate the molecular mechanism by which STING regulates NETs formation, we analyzed RNA sequencing data and identified seven genes associated with NETs formation that were significantly regulated by STING:*itgam, c3, casp4, fcgr1, fcgr4, fpr1, fpr2, tlr2*, and *mapk13* (**Supplementary Figure 1C**). Among these, *itgam* (encoding CD11b), an integrin gene, emerged as a key player. Previous studies have shown that depletion of maternal CD11b^+^ myeloid cells caused preterm birth and neonatal death, underscoring the critical role of integrins in implantation and inflammatory responses (37). CD11b is a heterodimeric partner of ITGB2, forming the macrophage-1 antigen receptor (MAC-1), which has been implicated in LPS-induced NETs formation. To examine the effects of STING on the expression of CD11b, an immunochemistry assay was performed the CD11b proteins. The results revealed a significantly downregulated of CD11b protein levels in STING-deficient mice compared to their WT counterparts (**Figures 4A, B**), suggesting that STING signaling is essential for maintaining CD11b expression in the endometrium. To further validate this finding, the STING inhibitor H-151 (1 μM) was used to inhibit STING playing functions and assessed CD11b expression. Consistent with the IHC results, pretreatment with H-151 led to a marked reduction in CD11b levels (**Figures 4C, D**), confirming that STING regulated CD11b expression in the endometrium.

To identify the key transcriptional regulators of *itgam*, we utilized the JASPAR database to predict transcription factors (TFs) that could bind to the *ITGAM* promoter. A total of 134 transcription factors were identified as potential regulators of *itgam* transcription (**Supplementary Figure 2A**). Through Venn analysis comparing these TFs with differentially expressed genes from STING transcriptome sequencing data, we narrowed down the list to three downregulated candidate TFs: IRF7, VDR and CEBPB (**Supplementary Figure 2B**). Binding sites predictions using the JASPAR database confirmed the presence of potential binding sites for these three transcription factors within the *itgam* promoter region (**Supplementary Figure 2C**), suggesting their direct regulator roles. To validate the involvement of these TFs in *itgam* regulation, the mRNA expression levels of *irf7*, *vdr* and *cebpb* in WT and STING-deficient mice was determined by qRT-PCR analysis. IRF7 mRNA expression levels were markedly upregulated in the WT LPS-treated group but significantly reduced in STING-deficient group (**Figure 4E**). Similarly, the mRNA levels of *vdr* and *cebpb* were also elevated in the WT LPS-induced group (**Supplementary Figure 2D–E**). Given that IRF7 has been strongly associated with the abundance of immune cells, including B cells, neutrophils and dendritic cells, we further investigated its protein expression in endometrial tissues. Western blotting analysis revealed a significantly downregulation of IRF7 protein in STING-deficient mice compared to WT controls (**Figures 4F, G**). In contrast, CEBPB protein levels showed no significant changes between the two groups (**Figure 4H**), suggesting that IRF7 is the primary TF regulated by STING in endometrium.

To further confirm the regulatory relationship between STING and IRF7, the STING inhibitor H-151were used to inhibit STING activation. Consistent with our earlier findings, IRF7 expression was significantly decreased in the H-151 treatment group (**Figures 4I, J**), demonstrating that STING activation is required for IRF7 upregulation. To establish a direct link between IRF7 and *itgam* expression, we overexpression IRF7 in Ishikawa cells and mouse primary endometrial cells. The analyses of qRT-PCR and Western blotting revealed that IRF7 overexpression led to a significant increase in *itgam* mRNA levels (**Figure 4K**) and CD11b protein expression (**Figures 4L, M**; **Supplementary Figure 2F–G**), confirming that IRF7 positively regulates *itgam* transcription and CD11b expression. To investigate whether STING directly activates neutrophils CD11b expression, we treated isolated bone marrow neutrophils with STING inhibitor C-176 and measured neutrophil CD11b expression using multicolor flow cytometry. The data demonstrated that C-176 significantly downregulated neutrophil CD11b expression (**Figures 4N, O**).

### STING-IRF7 signaling activated LCN2 in endometrium to drive ICAM-1 expression

**Figure 5:**
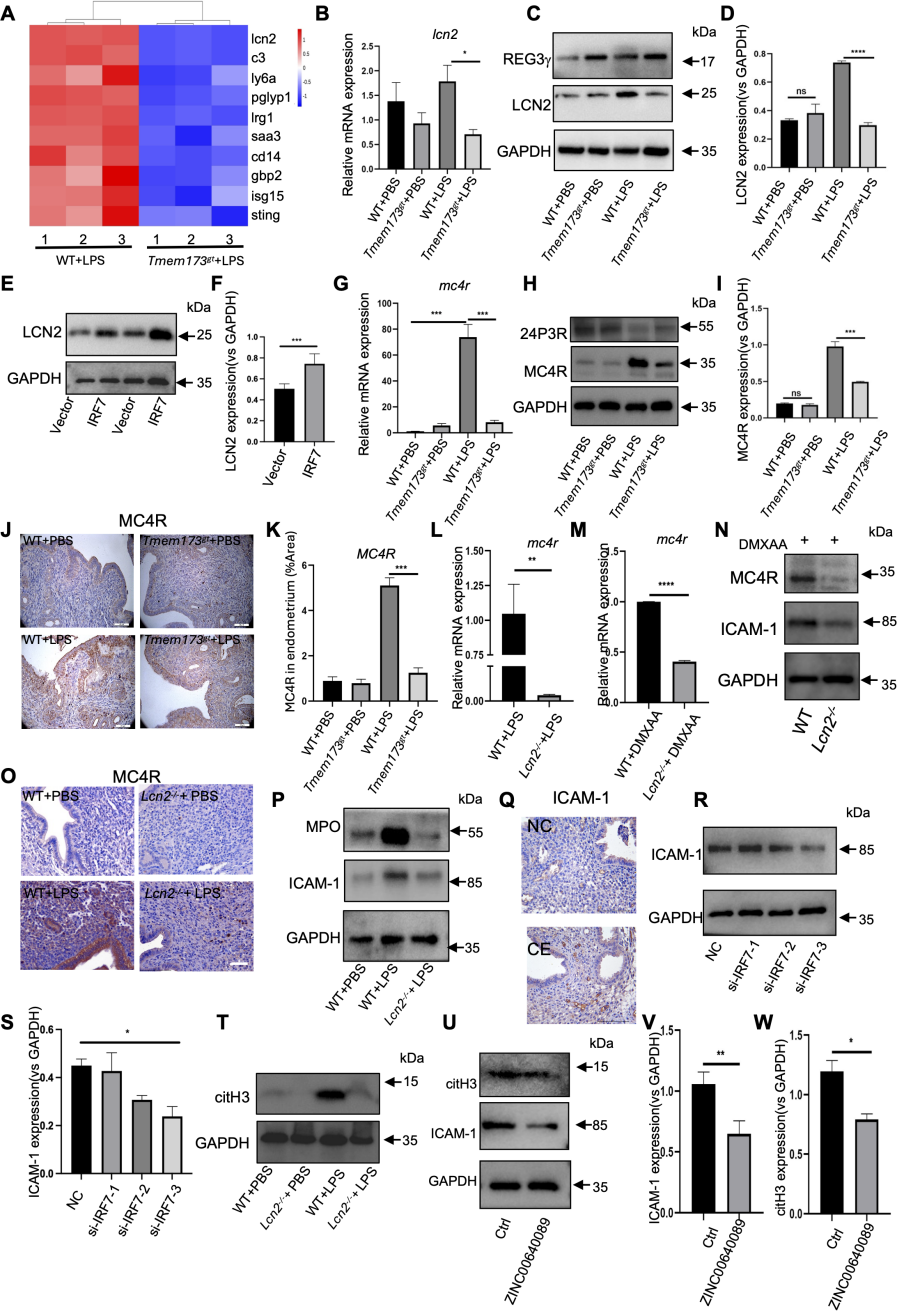
ICAM-1 was regulated by STING-IRF7-LCN2 axis in endometrium. **(A)** Heatmap of the first 10 DEGs from STING transcriptome. **(B)** Quantitative mRNA expression of *lcn2* in LPS stimulated *Tmem173^gt^* mice (n=6). One-way ANOVA test was applied with **P<0.01 (WT vs. *Tmem173^gt^* in LPS group). **(C)** Representative immunoblots of LCN2 in LPS-stimulated STING-deficient mice (*Tmem173^gt^*) endometrium tissues, compared with WT-LPS stimulated mice. (n=5). **(D)** Quantification of the amount of LCN2 in the four groups (n=4). One-way ANOVA test was applied with ****P<0.0001.**(E)** Representative immunoblots of LCN2 in EECs transfected with IRF7 for 48 (h) **(F)** Quantification of the amount of LCN2 in the two groups (n=4). Student’s *t* test was applied with ***P<0.001.**(G)** Quantitative mRNA expression of *mc4r* in LPS stimulated *Tmem173^gt^* mice (n=6). One-way ANOVA test was applied with ***P<0.001 (WT vs. *Tmem173^gt^* in LPS group). **(H)** Representative immunoblots of MC4R in LPS-stimulated STING-deficient mice (*Tmem173^gt^*) endometrium tissues, compared with WT-LPS stimulated mice. (n=5). **(I)** Quantification of the amount of MC4R in the four groups (n=4). One-way ANOVA test was applied with ****P<0.0001. **(J)** Representative immunohistochemical staining of MC4R in endometrial tissues of WT or STING-deficient mice (*Tmem173^gt^*) stimulated with LPS for 24 h at 400 × magnification (scale bar = 25 μm). **(K)** The area of MC4R were quantified (n=6). One-way ANOVA test was applied with **P<0.01 (WT vs. *Tmem173^gt^* in LPS group). **(L)** Quantitative mRNA expression of *mc4r* in LPS stimulated *Lcn2^-/-^* mice (n=6). Student’s *t* test was applied with **P<0.01. **(M)** Quantitative mRNA expression of *mc4r* in DMXAA stimulated *Lcn2^-/-^* mice (n=6). Student’s *t* test was applied with ****P<0.0001. **(N)** Representative immunoblots of MC4R and ICAM-1 in DMXAA stimulated *Lcn2^-/-^* mice endometrium tissues, compared with WT-LPS stimulated mice. (n=5). **(O)** Representative immunohistochemical staining of MC4R in endometrial tissues of WT or *Lcn2^-/-^* mice stimulated with LPS for 24 h at 400 × magnification (scale bar = 25 μm). **(P)** Representative immunoblots of MPO and ICAM-1 in endometrial tissues of WT or *Lcn2^-/-^* mice treated with LPS for 24 (h) **(Q)** Representative immunohistochemical staining of ICAM-1 in endometrial tissues of chronic endometritis or control at 400 × magnification (scale bar = 25 μm). **(R)** Representative immunoblots of ICAM-1 in EECs transfection with si-IRF7 (1,2,3). **(S)** Quantification of the amount of ICAM-1 in the four groups (n=4). One-way ANOVA test was applied with *P<0.05.**(T)** Representative immunoblots of citH3 in endometrial tissues of WT or *Lcn2^-/-^* mice treated with LPS for 24 (h) **(U)** Representative immunoblots of citH3 and ICAM-1 in EECs-neutrophils coculture system with the stimulation of ZINC00640089. **(V)** Quantification of the amount of ICAM-1 in the two groups (n=5) (ctrl vs. ZINC00640089). Student’s *t* test was applied with **P<0.01. **(W)** Quantification of the amount of citH3 in the four groups (n=5) (ctrl vs. ZINC00640089). Student’s *t* test was applied with *P<0.05.

Based on the transcriptomic profiling of uterine tissues analysis, 1449 upregulated and 1677 downregulated genes (|log2 (Fc)| > 1 and adj. *P*<0.05) were identified in STING-deficient mice versus WT controls. Among the top 10 downregulated expression genes (**Figure 5A**), LCN2 exhibited the most pronounced reduction in expression, prompting further investigation into its regulation by STING. To assess STING-dependent regulation of LCN2 in the endometrium, we performed qRT-PCR and Western blotting. Both LCN2 mRNA and protein levels were robustly induced in LPS-treated WT mice but markedly attenuated in STING-deficient mice (**Figures 5B–D**). Previous study showed that IRF7 promoted LCN2 transcription (38), here, we transfection of IRF7 expression plasmid in EEC cells and detected the expression of LCN2 (**Figure 5E**). The result showed that the expression of LCN2 was promoted by IRF7, indicating that STING-IRF7 axis contributed to LCN2 expression (**Figure 5F**). We next interrogated endometrial expression of LCN2 receptors-24p3R, megalin and MC4R. While 24p3R and megalin mRNA levels decreased in STING-deficient mice (**Figure 5G**; **Supplementary Figure 2H-I**), MC4R expression was most significantly reduced in STING-deficient mice. The results of Western blotting and immunohistochemistry confirmed diminished MC4R protein in STING-deficient endometria following LPS challenge (**Figures 5H–K**), establishing the LCN2-MC4R axis as a STING-IRF7 regulated pathway. To define the functional role of LCN2 in endometritis, LPS was administered to LCN2-knockout (*Lcn2^-/-^*) mice. Quantitative PCR revealed the mRNA levels of *mc4r* was decreased both in LPS stimulated *Lcn2^-/-^* mice (**Figure 5L**), and STING agonist DMXAA stimulated *Lcn2^-/-^* mice compared with WT mice (**Figure 5M**). Immunoblotting of MC4R confirmed a negative effect of DMXAA in mice (**Figure 5N**). Immunohistochemical staining of MC4R in endometrial tissues of *Lcn2^-/-^* mice treated with LPS was also decreased compared with WT mice (**Figure 5O**). Myeloperoxidase (MPO) levels, indicative of neutrophil infiltration, were reduced in *Lcn2^-/-^* versus WT mice post-LPS treatment (**Figure 5P**). Concurrently, LPS-induced expression of ICAM-1 on the cell surface of epithelial interacting with neutrophils was attenuated in *Lcn2^-/-^* versus WT mice post-LPS treatment (**Figure 5P**). Immunohistochemistry analysis further revealed elevated ICAM-1 expression in endometrial biopsies form patients with chronic endometritis (**Figure 5Q**). Immunoblotting found a significant downregulation of ICAM-1 protein expression in EEC cells transfected with si-IRF7 (**Figures 5R,S**). Immunoblotting of ICAM-1 confirmed a negative effect of DMXAA in *Lcn2^-/-^* mice (**Figure 5N**). Furthermore, citH3, a marker of NETs formation, was marked decreased in LPS-stimulated *Lcn2^-/-^* mice (**Figure 5T**). Blocking LCN2 with LCN2 inhibitor ZINC00640089 in epithelial-neutrophil cocultures significantly downregulated ICAM-1 and citH3 expression (**Figures 5U–W**). These data demonstrate that STING governs chronic endometrial inflammation through the LCN2-MC4R axis, which drives ICAM-1 mediated neutrophil recruitment and NETs formation.

Additionally, IL-17C is an epithelial-derived cytokine that is involved in recruitment neutrophils with high NETosis activity and is expressed earlier in infection than other IL-17 cytokine family members (39, 40). Since IL-17C upregulates the expression of a variety of other inflammatory mediators and chemokines, future investigations into the possibility that blocking IL-17C activity might limit chronic inflammation (41, 42). KEGG Pathway enrichment analysis identified significant dysregulation of the IL-17 signaling cascade in STING-deficient endometria (**Figure 2C**). STING-dependent transcriptional control was observed for 27 IL-17-associated genes, including IL-17C, CXCL2, and CXCL5. Consistent with this, qRT-PCR revealed marked downregulation of *il-17c, cxcl2*, and *cxcl5* in LPS-challenged STING-deficient mice compared to WT controls (**Supplementary Figure 3A–C**). Immunofluorescence further corroborated reduced IL-17 protein levels in STING-deficient endometria post-LPS challenge (**Supplementary Figure 3D**). concomitantly, immunohistochemical staining for Ly-6G, the neutrophil marker, demonstrated significantly attenuated neutrophil infiltration in STING-deficient mice following LPS exposure (**Supplementary Figure 3E**). These results indicated that IL-17C enhanced endometrium epithelial expression chemokines CXCL2/CXCL5 that attracted neutrophils across STING signaling.

### Deficiency of STING partially restored embryo receptivity during chronic inflammatory condition in mice

**Figure 6:**
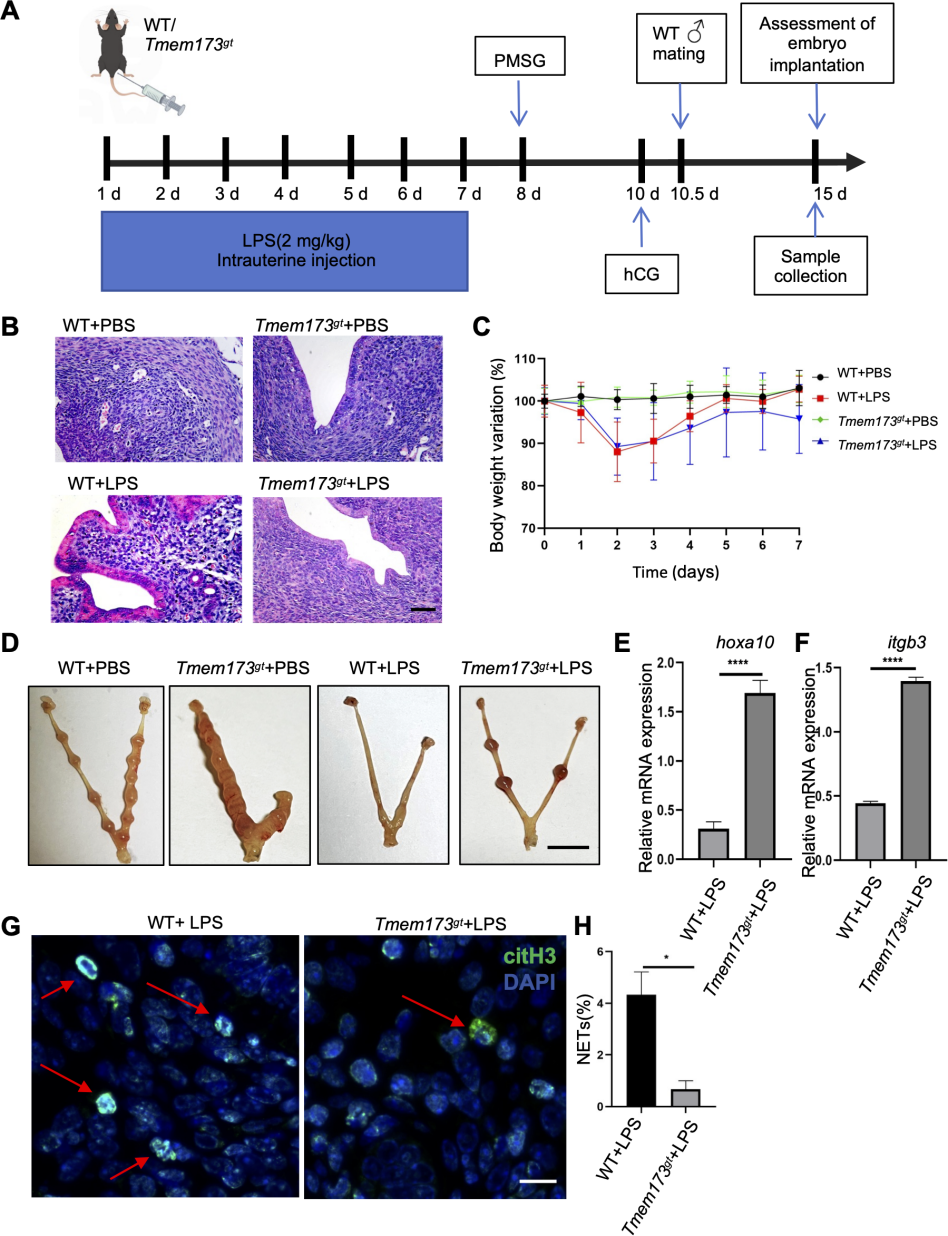
Deficiency of STING partially restored embryo implantation during chronic endometritis in mice. **(A)** Schematic illustration of animal experiments. **(B)** Representative H&E staining in the endometrial tissues of WT or STING-deficient mice in the PBS or LPS 7.5 days post infection (dpi) groups at 400 × magnification (scale bar = 25 μm). **(C)** Body weight variation of WT and *Tmem173^gt^* mice with or without the stimulation of LPS (n = 6). **(D)** Morphology of embryos at gestational 7.5 days in four groups. **(E)**Quantification of mRNA expression of *hoxa10* in the two groups (n=5) (WT vs. *Tmem173^gt^* in LPS group). Student’s *t* test was applied with ****P<0.0001. **(F)** Quantification mRNA expression of *itgb3* in the four groups (n=5) (WT vs. *Tmem173^gt^* in LPS group). Student’s *t* test was applied with ****P<0.0001. **(G)** Representative immunohistochemistry staining of citH3 in in WT or STING-deficient mice endometrial tissues of chronic endometritis at 400 × magnification (scale bar = 25 μm). The staining color of citH3 was green. Arrows indicate NETs. The DNA was staining with DAPI. **(H)** Quantification of the percentage of citH3-positive NETs in in LPS stimulated WT or STING-deficient mice endometrial tissues. (n=5) (WT vs. *Tmem173^gt^* in LPS group). Student’s *t* test was applied with *P<0.05.

To investigate the impact of STING on embryo receptivity in chronic endometritis, another chronic endometritis mice model was conducted that WT and STING-deficient mice were subjected to intrauterine LPS perfusion once daily for seven consecutive days (**Figure 6A**). After 7.5 days, samples were collected and analyzed for inflammation. H&E staining revealed severe epithelial detachment and glandular apoptosis in LPS-treated WT mice, phenotypes that were substantially attenuated in STING-deficient endometria (**Figure 6B**). The body weight variation has no significant differences in WT and *Tmem173^gt^* mice treatment with PBS or LPS (**Figure 6C**). Strikingly, LPS triggered complete pregnancy failure in WT mice at 5 days post conception, whereas STING deficiency restored implantation sites (**Figure 6D**), directly linking STING activity to inflammation-driven reproductive dysfunction. We evaluated endometrial receptivity via the transcription factor homeobox A10 (HOXA10) and its downstream effector integrin beta 3(ITGβ3), critical for embryo implantation. Despite LPS-induced inflammation, STING deficiency partially rescued *hoxa10 and itgb3* mRNA expression during gestation (**Figures 6E, F**), suggesting restored receptivity. Furthermore, to dissect the role of STING in NETs formation, the expression of the NETs-related proteins citH3 and ELA2 was also detected by immunohistochemical staining in this chronic endometritis mouse model. We found that for more time stimulated with LPS in LPS-challenged WT endometria also increase in citH3 and ELA2 and NETs formation (**Figures 6G, H**; **Supplementary Figure 4A**), both of which were blunted in STING-deficient mice, indicating STING-dependent NETs regulation.

### NETs formation was related to chronic endometritis

**Figure 7:**
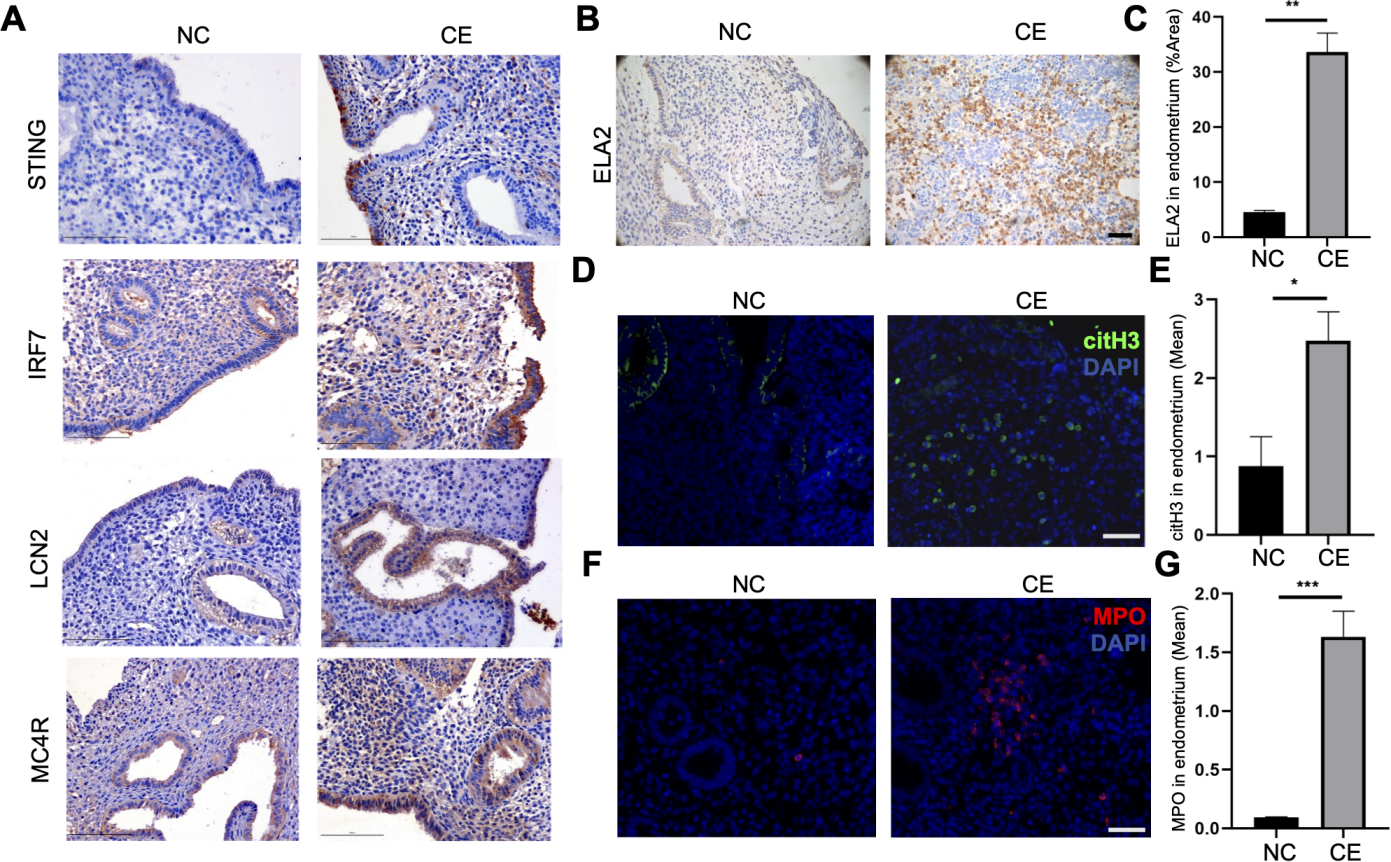
Neutrophil NETs formation in chronic endometritis patients. **(A)** Representative immunohistochemical staining of STING, IRF7, LCN2 and MC4R in endometrial tissues of chronic endometritis or control at 400 × magnification (scale bar = 100 μm). **(B)** Representative immunohistochemical staining of ELA2 in endometrial tissues of chronic endometritis or control at 400 × magnification (scale bar = 25 μm). **(C)** The area of ELA2 was quantified (n=6). Student’s *t* test was applied with **P<0.01. **(D)** Representative immunofluorescence image of citH3 in endometrial tissues of chronic endometritis or control at 400 × magnification (scale bar = 25 μm). The staining color of citH3 was green. The DNA was staining with DAPI. **(E)** The mean of citH3 were quantified (n=6). Student’s *t* test was applied with *P<0.05. **(F)** Representative immunofluorescence image of MPO in endometrial tissues of chronic endometritis or control at 400 × magnification (scale bar = 25 μm). The staining color of MPO was red. The DNA was staining with DAPI. **(G)** The mean of MPO were quantified (n=6). Student’s *t* test was applied with ***P<0.001.

To demonstrate the STING-IRF7-LCN2 axis formation in patients with chronic endometritis, the immunohistochemical staining of STING, IRF7, LCN2 and MC4R were detected, and the results showed these expressions had accumulated in the endometrium of chronic endometritis patients, which supports that STING-IRF7-LCN2 axis was activated during endometrial inflammation (**Figure 7A**; **Supplementary Figure 5A–D**). NETs are composed of citrullinated histone H3 (citH3), elastase 2 (ELA2), and myeloperoxidase (MPO). To investigate NETs formation in the uterine microenvironment during chronic endometritis, two hallmark NETs components ELA2 (**Figures 7B, C**) and MPO (**Supplementary Figure 5E–F**) were prominently detected via IHC staining in endometrial tissues of chronic endometritis patients. Immunofluorescence analysis further revealed minimal citH3^+^ cells in normal endometrial tissues, whereas citH3^+^ signals were significantly elevated in chronic endometritis (**Figures 7D, E**), consistent with enhanced NETosis. Similarly, immunofluorescence staining with MPO-expressing neutrophil infiltration was scarce in controls but markedly increased in chronic endometritis uteri (**Figures 7F, G**), further corroborating aberrant neutrophil activation and NETs deposition in endometritis progression.

## Discussion

While chronic endometritis is characterized by dysbiotic endometrial microbiota and CD138+ plasma cell infiltration (43, 44), the molecular drivers of immune hyperactivation have remained elusive. Our study uncovers STING-a canonical cytosolic DNA sensor-as a previously unrecognized orchestrator of endometrial inflammation. We demonstrate that STING activation in CE propagates a self-amplifying inflammatory cascade: by upregulating pro-inflammatory cytokines CXCL2/5, recruiting neutrophils via ICAM-1 and inducing NETs, STING creates a pathological microenvironment that exacerbates epithelial damage and impairs endometrial repair. Crucially, genetic ablation of STING abrogates these effects, rescuing both histological and functional endpoints of endometrial integrity. The attenuation of these effects in STING-deficient mice highlights the potential of targeting STING signaling as a therapeutic strategy for managing chronic endometritis and associated inflammatory conditions.

Although NETs are essential for pathogen clearance, their dysregulated formation-marked by excessive MPO, ROS, and cytotoxic chromatin release-has emerged as a double-edged sword, driving tissue damage and immune dysfunction in sepsis, autoimmunity and cancer (45–47). Our study now positions aberrant NETosis as a pivotal mechanism bridging acute to chronic endometritis, a transition previously attributed solely to microbial persistence. By integrating clinical observations with mechanistic dissection in murine models and *ex vivo* stimulation of neutrophils with STING agonist or inhibitor, we establish that STING-IRF7 signaling acts as a master switch for endometrial NETosis through transcriptional control of itgam (CD11b), a neutrophil activation marker critical for NETs release (48–50). In the present study, we uncover a previously unrecognized axis wherein STING activation in endometrial epithelial cells-potentially triggered by microbiota-derived DNA or DAMPs-upregulates IRF7, which directly binds the itgam promoter to license CD11b expression. This cascade converts neutrophils into a hyperactivated stage, enabling rampant NETosis that perpetuates inflammation. Strikingly, STING deficiency not only suppressed CD11b but also abrogated downstream NETs markers (MPO, cit-H3 and elastase), revealing a linear pathway from DNA sensing to neutrophil-mediated endometrial injury. While NETs are canonically antimicrobial, their pathological persistence in CE aligns with recent paradigms in rheumatoid arthritis, where NETs debris fuels autoantibody production (51). Our findings extend this concept to reproductive inflammation and suggest that NETs-derived MPO may disrupt endometrial repair by oxidizing receptivity factors (e.g., HOXA10).

Combining with *in vivo* LCN2 knockout mice and *in vitro* blocking LCN2 in epithelial–neutrophil cocultures definitively identify LCN2 as a mediator driving ICAM-1 expression and NETosis formation. Our study delineates a dual mechanism by which STING propagates endometrial inflammation and reproductive dysfunction: (1) via the LCN2-MC4R-ICAM1 axis, it orchestrates neutrophil infiltration and NETosis, and (2) through suppression of HOXA10-mediated endometrial receptivity, it directly couples innate immune hyperactivation to implantation failure. This bifurcated pathway positions STING as a master regulator of sterile inflammation in chronic endometritis, diverging from its canonical role in antiviral defense. Our observation that STING deficiency rescues implantation sites aligns with studies showing that aberrant neutrophil activity and NETs impair endometrium function (52–54). While STING is recognized for epithelial repair in colitis via STAT3-dependent restitution colitis (55), its proinflammatory role in CE underscores tissue-specific signaling outcomes. Here, STING activation in endometrial stromal cells-likely by endogenous DAMPs rather than pathogens-drives IL-17C and CXCL2/5 production, creating a chemotactic gradient for neutrophils. These neutrophils, licensed by ICAM-1 adhesion, release NETs laden with MPO and citrullinated histones, which we show directly degrade HOXA10/ITGβ3, critical mediators of embryo adhesion. To our knowledge, this is the first demonstration that NETs mechanistically impair endometrial receptivity, a paradigm shift linking neutrophil dysregulation to infertility. Paradoxically, the microbial role of STING in infection contrasts its pathological function in CE. This dichotomy may hinge on spatiotemporal activation dynamics: transient STING signaling eliminates pathogens, whereas chronic activation (as in CE) exhausts reparative responses.

Emerging evidence implicates STING as a sentinel of infection-driven endometrial pathology, with mitochondrial DNA leakage activating this pathway to exacerbate LPS-induced inflammation (56) and chronic ectopic lesions exhibiting STING-dependent lymphocyte infiltration (57, 58). Our prior clinical work further established elevated STING activation as a hallmark of chronic endometritis (59). However, the functional interplay between STING signaling and reproductive outcomes during endometrial infection has remained unexplored. Here, we resolve this gap by demonstrating that STING activation directly couples infection-induced inflammation to embryo implantation failure-a mechanistic link with profound implications for inflammatory infertility. This finding challenges the prevailing view of STING as a purely defensive pathway, instead positioning it as a double-edged modulator of endometrial homeostasis: while acute STING activation may combat pathogens, its chronic engagement-as seen in clinical CE-disrupts the delicate balance between immune vigilance and receptivity.

While our LPS-induced murine model recapitulates chronic endometrial inflammation, a key limitation lies in its inability to fully mirror the chronic, low-grade immune dysregulation characteristic of human CE. To bridge this translational gap, patient-derived endometrial organoids-retaining native stromal-epithelial-immune interactions-should be leveraged to dissect the role of STING in sustained sterile inflammation. Parallel studies in non-infectious CE model (e.g., autoimmune-driven or metabolic stress) could further delineate STING’s ligand-specific contributions. Notably, the interplay between endometrial microbiota and STING remains an open frontier. While *Gardnerella*, a CE-associated pathobiont, is implicated in NETosis (60), whether it directly activates STING via genomic DNA or cyclic dinucleotides requires rigorous testing using gnotobiotic models or bacterial-STING co-culture systems. Such work could reveal microbiome-STING crosstalk as a therapeutic node, particularly in CE subtypes resistant to antibiotic eradication. Equally critical is defining the cellular niche governing the role of STING in reproductive effects. Does STING in stromal cells drive immune recruitment via chemokines, while epithelial STING directly impairs receptivity? Sing-cell and spatial transcriptomics of CE patient endometrial could resolve this, mapping STING/IRF7 activity to specific compartments and identifying neutrophil-stromal crosstalk hubs enriched in NET-promoting factors (e.g., S100A8/9). Conditional STING knockout models (e.g., *Lgr5+*-epithelial vs. *CD45+*-immune) would further isolate cell type-specific contributions.

In conclusion, our study elucidates a self-reinforcing inflammatory circuit in chronic endometritis, wherein STING-IRF7 activation drives dual pathogenic cascades:1) transcriptional upregulation of CD11b to amplify neutrophil recruitment, and 2) LCN2-MC4R-mediated ICAM-1 adhesion and NETosis, collectively fostering a microenvironment that suppresses HOXA10-the master architect of endometrial receptivity. This mechanistic framework explains the enigmatic prevalence of occult CE in idiopathic infertility, positioning aberrant STING signaling as a linchpin between immune hyperactivation and reproductive failure. Critically, we redefined NETs not merely as antimicrobial effectors but as active perpetrators of endometrial dysfunction in chronic inflammation. By coupling neutrophil-driven tissue injury to receptivity loss, NETs create a “fertility trap” that perpetuates CE pathology. Our findings further nominate STING as a keystone target in inflammatory infertility: pharmacological inhibition STING (e.g., H-151) could disrupt this vicious cycle while sparing systemic immunity-a pivotal advantage for women balancing infection control and fertility preservation.

## Data Availability

The original contributions presented in the study are publicly available. This data can be found here: NCBO GEO repository, accession GSE314991.
