# Correction: Sustained STING-IRF7 signaling aggravates LPS-induced endometrial inflammation via excessive neutrophil extracellular traps generation

**DOI:** 10.3389/fimmu.2026.1839224

**Published:** 2026-04-01

**Authors:** Min Chu, Ding Ma, Zhan Song, Li Liang, Fengjuan Xing, Hongchu Bao

**Affiliations:** 1 Reproductive Medicine Center, The Affiliated Yantai Yuhuangding Hospital of Qingdao University, Yantai, China; 2 Shandong Provincial Key Medical and Health Laboratory of Reproductive Health and Genetics (Yantai Yuhuangding Hospital), Yantai, China; 3 Central Laboratory, The Affiliated Yantai Yuhuangding Hospital of Qingdao University, Yantai, China; 4 Department of Pathology, The Affiliated Yantai Yuhuangding Hospital of Qingdao University, Yantai, China

**Keywords:** CD11b, chronic endometritis, IRF7, neutrophil extracellular traps, STING

An incorrect **Funding** statement was provided. The correct **Funding** statement reads:

“The author(s) declared that financial support was received for this work and/or its publication. This study was supported by Shandong Provincial Natural Science Foundation (ZR2022QH370) and the Ministry of Health of China, Wu Jieping Medical Foundation (320.6750.2022-06-40) and the Project for Enhancing the Innovation Capability of Technology-based Small and Medium-sized Enterprises in Shandong Province (2023TSGC0921).”

The original version of this article has been updated.

