# Supplementary material for: Sustained STING-IRF7 signaling aggravates LPS-induced endometrial inflammation via excessive neutrophil extracellular traps generation
Source: Front Immunol. 2026 Jan 9;16:1671848. doi: 10.3389/fimmu.2025.1671848 (PMC12827698; doi:10.3389/fimmu.2025.1671848)
Supplement: Supplementary file 2 [file SupplementaryFile1.pdf]

# Supplementary Figures:

## Supplementary Figure 1

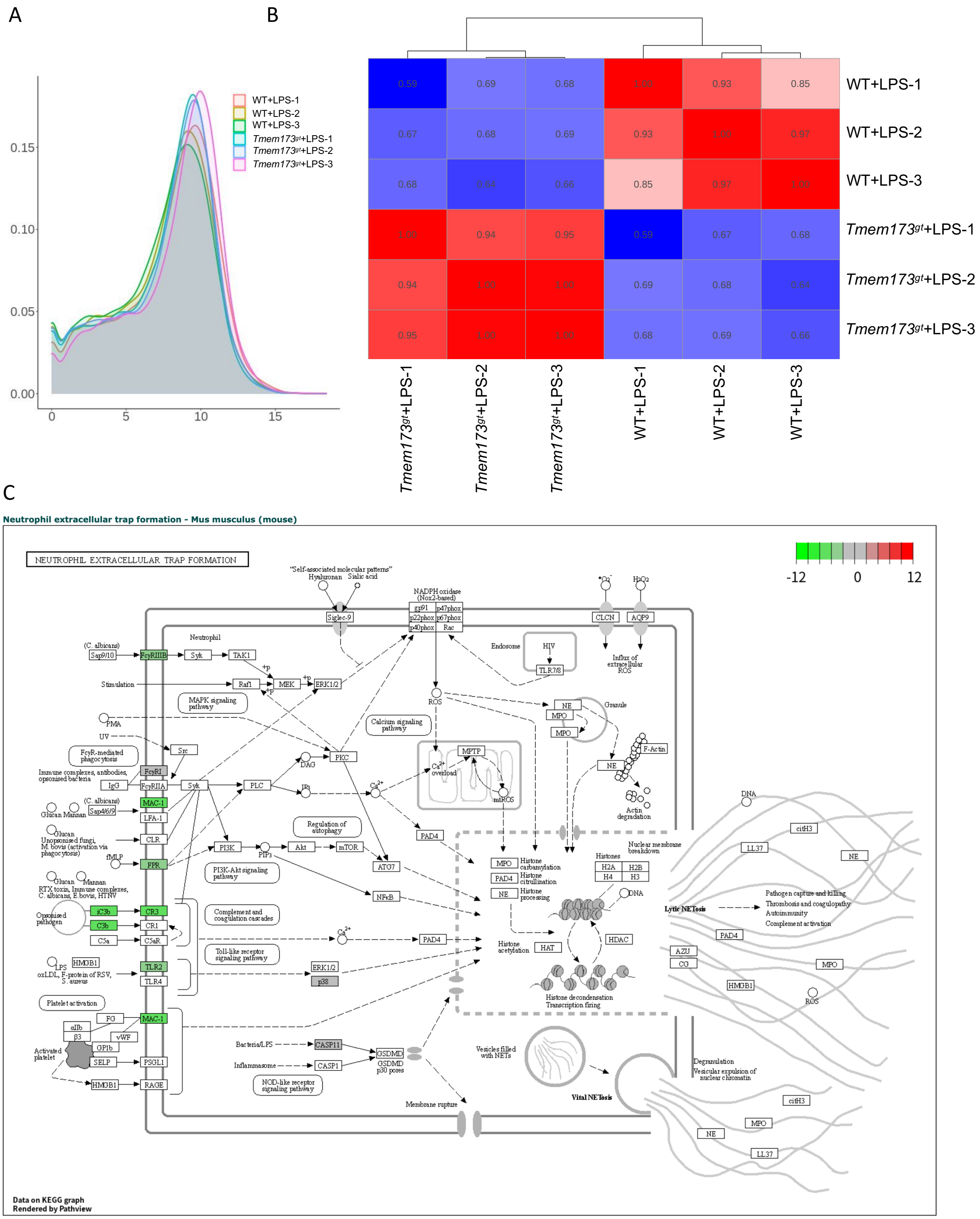

**Supplementary Figure 1. Genes expressing about NETs regulated by STING in endometrium.** (A) The transcription sequence of STING-deficiency mouse endometritis tissues. Distribution of samples expression. The higher the probability density distribution of gene expression and the diagram for log2 FPKM abscissa, the higher amount of gene expression. FPKM: fragments per kilobase per million mapped reads. (B) Heatmap of genes expressed across the WT and STING-deficient mice (Tmem173gt) infected by LPS for 24 h group. Each row represents one gene, and each column represents one sample. (C) KEGG annotation analysis of differential expression genes expressed in neutrophils extracellular traps.

# Supplementary Figure 2

**A**

| | | | | | | | | | | | |
|---|---|---|---|---|---|---|---|---|---|---|---|
| Il4r | Tbx1 | Tsc22d4 | Kmt2d | Zmat3 | Sohlh2 | **Vdr** | Mafg | Kdm2a | Pbx1 | Hoxc13 | Nrf1 |
| Foxa2 | Nr4a1 | Tfap2a | Adnp | Prox1 | Myod1 | Yy1 | Hipk2 | Cpeb1 | Mapk9 | Xbp1 | Ets2 |
| Eed | Neurod1 | Actl6a | Plagl2 | Phf8 | Junb | Esr1 | Snai1 | Wt1 | Brca1 | Foxk1 | |
| Fubp1 | Tbx20 | Irf6 | Rest | Sohlh1 | Zbp1 | Mecp2 | Mef2b | Jmjd6 | Arid1a | Erg | |
| Bptf | Cebpa | Gata4 | Crebbp | Tet1 | Pparg | Nsd2 | Sox9 | Sox21 | Sall1 | Gata1 | |
| p63 | Lmx1b | Stat6 | Klf6 | Padi2 | Nelfb | Arid2 | Hnrnpk | Hnf1a | Myb | Cebpe | |
| Nr2f2 | Sox11 | Prmt1 | Gfi1 | Med19 | Pcgf6 | Cbx2 | Arid1b | Bhlha9 | Gata3 | Siah2 | |
| Mkl1 | Runx1 | Nfix | Hsf4 | Brpf1 | Jarid2 | Gtf2ird1 | Sox17 | Mkx | E2f4 | Twist1 | |
| Lin28a | Irf8 | Spdef | Batf2 | Mef2c | Gata2 | Mbd1 | **Cebpb** | Mef2d | Satb2 | Dnmt3l | |
| Dnmt3b | Sirt3 | Pdx1 | Bcl11b | Tet2 | Bcl6 | Hmg20a | Stat3 | Ctnnb1 | Sirt6 | Klf17 | |
| Srf | Cbfb | Kat6a | **Irf7** | Meis1 | Prdm2 | Sall4 | Ahr | Mef2a | Atrx | Kat6b | |
| Setd2 | Bcl3 | Sp3 | Arhgap35 | Dmrt1 | Kdm5b | Ssrp1 | Atoh8 | Npas4 | Foxp1 | Elf5 | |

**B**

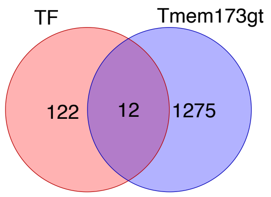


| | | | |
|---|---|---|---|
| Brca1 | Snai1 | Vdr | Ssrp1 |
| Irf6 | Prmt1 | Dnmt3b | Elf5 |
| Cebpb | Zbp1 | Pparg | Irf7 |

**C**

| log2FoldChange | pvalue | padj | Gene Symbol | description |
|---|---|---|---|---|
| -2.916692727 | 0.000282025 | 0.01118716 | *Irf7* | interferon regulatory factor 7 |
| -1.756159214 | 7.28E-07 | 0.000177792 | *Vdr* | vitamin D (1,25-dihydroxyvitamin D3) receptor |
| -2.014746944 | 0.000456625 | 0.015728179 | *Cebpb* | CCAAT/enhancer binding protein (C/EBP), beta |

**D** **E**

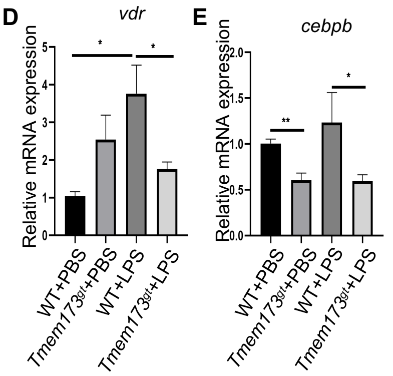


**F**

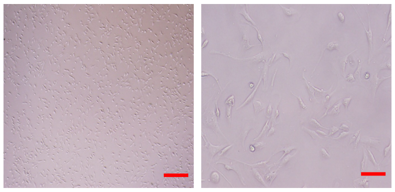

**G**

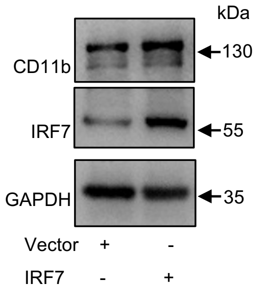


**H** **I**

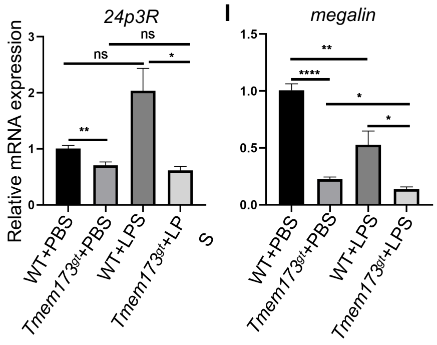

**Supplementary Figure 2. Screen genes between transcription factors and STING transcriptome sequencing.** (A) The transcription factors regulating itgam transcription were performed using KnockTF 2.0 website(https://bio.liclab.net/KnockTFv2/index.php). (B) Venn showed the common genes between these transcription factors and the differenced expressed genes in STING transcriptome sequencing. (C) The binding sites between differently expressed transcription factors and CD11b promoters were predicted by JASPAR website(http://jaspar.genereg.net/). (D) Quantitative mRNA expression of *vdr* in LPS stimulated Tmem173$^{gt}$ mice (n=6). One-way ANOVA test was applied with *$P < 0.05$ (WT vs. Tmem173$^{gt}$ in LPS group). (E) Quantitative mRNA expression of *cebpb* in LPS stimulated Tmem173$^{gt}$ mice (n=6). One-way ANOVA test was applied with *$P < 0.05$ (WT vs. Tmem173$^{gt}$ in LPS group). (F)The endometrial epithelial cells (EECs) were enzymatically isolated from the uterine tissues of the mice. (G) The protein expression of CD11b was detected by Western blotting with the transfection of IRF7 in mouse primary endometrium cells. (H) Quantitative mRNA expression of *24p3R* in LPS stimulated Tmem173$^{gt}$ mice (n=6). One-way ANOVA test was applied with ns (WT vs. Tmem173$^{gt}$ in LPS group). (I) Quantitative mRNA expression of *megalin* in LPS stimulated Tmem173$^{gt}$ mice (n=6). One-way ANOVA test was applied with *$P < 0.05$ (WT vs. Tmem173$^{gt}$ in LPS group).

Supplementary Figure 3

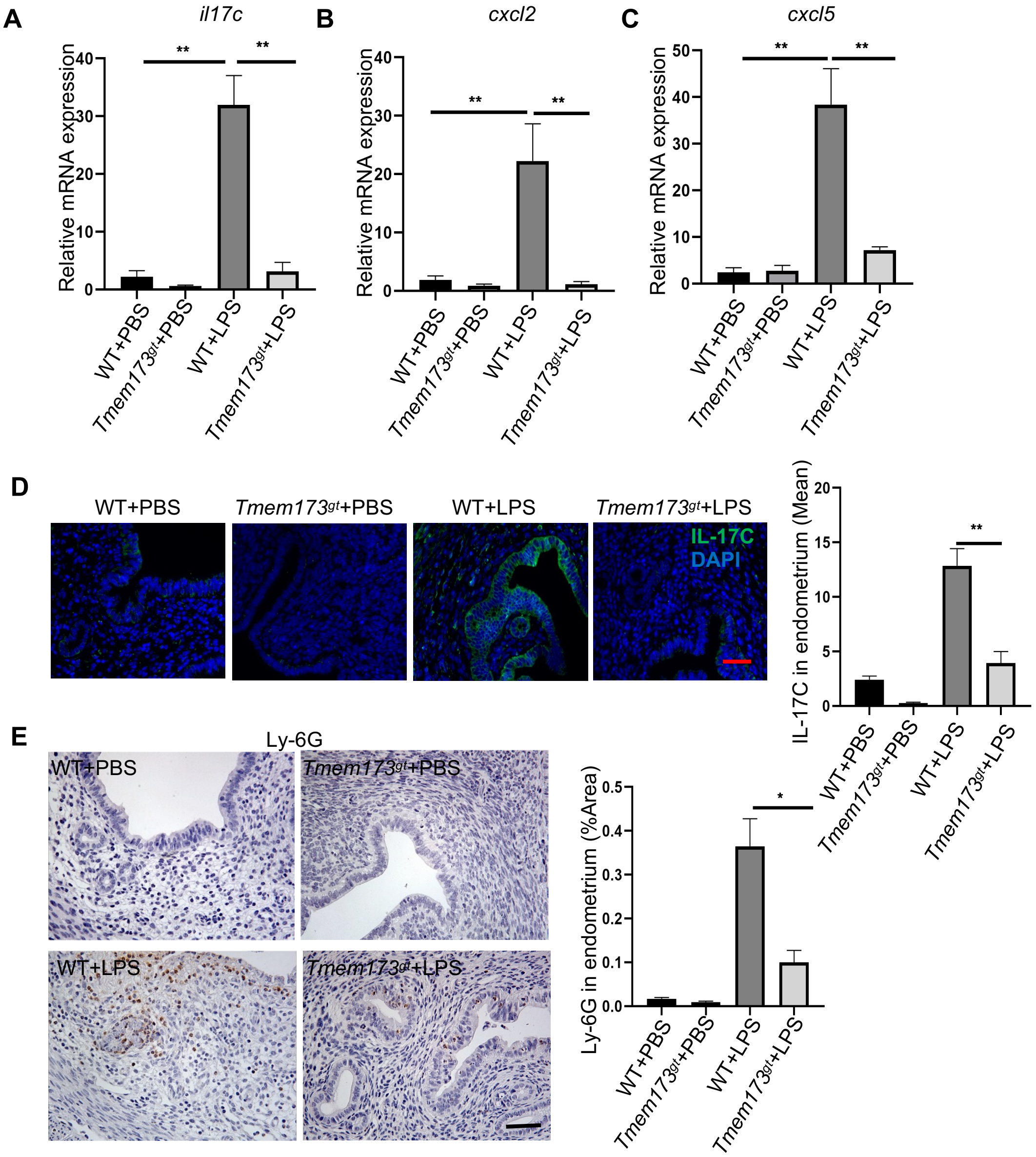


**Supplementary Figure 3. IL-17C enhancing epithelial expression chemokines CXCL2/CXCL5 that attracted neutrophils across STING signaling.** Graphs showed mRNA expressions of *il17c* (A), *cxcl2* (B) and *cxcl5* (C) in endometrial tissues across the WT or STING-deficient mice (Tmem173gt) infected by LPS detected by qRT-PCR (n=6). One-way ANOVA test was applied with $^{**}P < 0.01$ (WT vs. $Tmem173^{gt}$ in LPS group). (D) Immunofluorescence staining

of IL-17C in WT or STING-deficient mice (Tmem173gt) with LPS infected endometrium at 400 × magnification (n=5, scale bar = 25 mm). The staining color of IL-17C was green. The DNA was staining with DAPI. The mean of IL-17C were quantified. One-way ANOVA test was applied with $^{**}P < 0.01$ (WT vs. $Tmem173^{gt}$ in LPS group). (E) Immunohistochemical staining of Ly-6G in endometrium tissues of WT or STING-deficient mice (Tmem173gt) with LPS infected endometrium at 400 × magnification (n=5, scale bar = 25 mm). The area of Ly-6G were quantified. One-way ANOVA test was applied with $^{*}P < 0.05$ (WT vs. $Tmem173^{gt}$ in LPS group).

Supplementary Figure 4

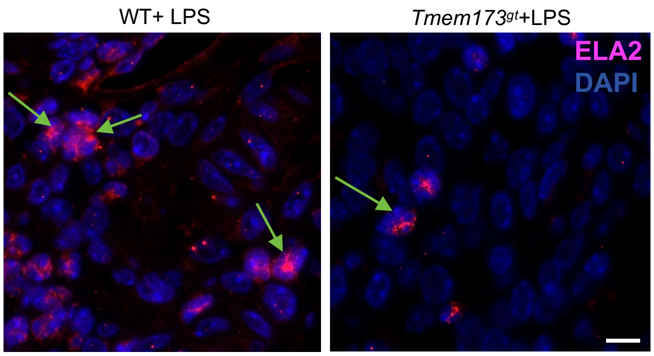


**Supplementary Figure 4. Immunofluorescence staining of ELA2 in mice.** Immunohistochemistry staining of ELA2 in WT or STING-deficient mice endometrial tissues of chronic endometritis at 400 × magnification (n=5, scale bar = 25 mm). The staining color of ELA2 was red. The DNA was staining with DAPI.

Supplementary Figure 5

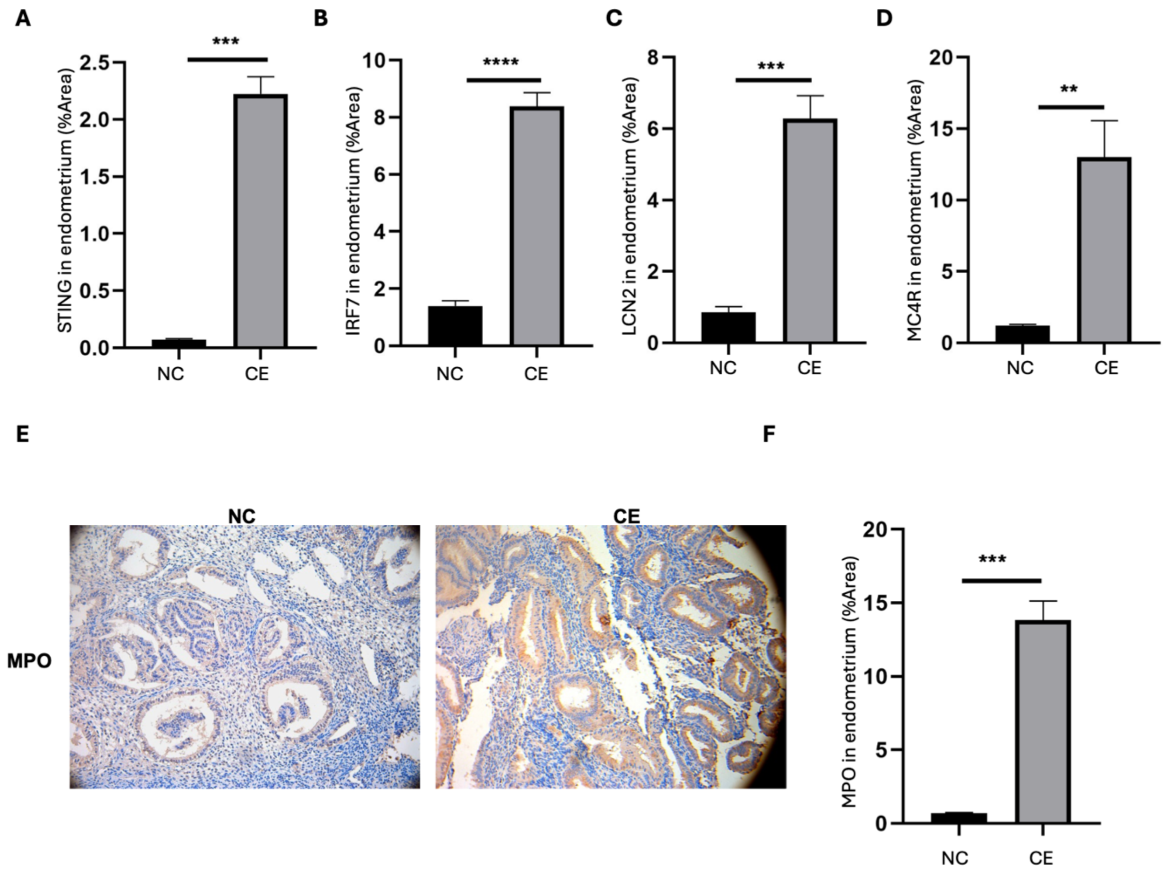


**Supplementary Figure 5. The NETs formation in chronic endometritis patients.** (A) The area of STING was quantified (n=6). Student's *t* test was applied with ***$P < 0.001$. (B) The area of IRF7 was quantified (n=6). Student's *t* test was applied with ****$P < 0.0001$. (C) The area of LCN2 was quantified (n=6). Student's *t* test was applied with ***$P < 0.001$. (D) The area of MC4R was quantified (n=6). Student's *t* test was applied with **$P < 0.01$. (E) Immunohistochemical staining of MPO in endometrial tissues of chronic endometritis or control at 100 × magnification (scale bar = 100 mm). (F) The area of MPO were quantified. (n=6). Student's *t* test was applied with ***$P < 0.001$.
